# Evaluation of upper limb perception after stroke with the new Affected Limb Perception Questionnaire (ALPQ): a study protocol

**DOI:** 10.1186/s12883-024-03648-6

**Published:** 2024-06-11

**Authors:** Stéphanie Konik, Valérie Beaud, Julia Fellrath, Isabella Martinelli, Eleonora Guanziroli, Franco Molteni, Michela Bassolino, Andrea Serino

**Affiliations:** 1 MySpace Lab, Department of Clinical Neurosciences, Lausanne University Hospital and University of Lausanne, Lausanne, Switzerland; 2 Service of Neuropsychology and Neurorehabilitation, Lausanne University Hospital and University of Lausanne, Lausanne, Switzerland; 3 Département Hospitalier, Institution de Lavigny, Lavigny, Switzerland; 4 Villa Beretta Rehabilitation Center, Ospedale Valduce, Costa Masnaga, Italy; 5 School of Health Sciences, Institute of Health, HES-SO Valais-Wallis, Sion, Switzerland; 6 Department of Brain and Behavioral Sciences, University of Pavia, Pavia, Italy; 7 The Sense, Innovation and Research Center, Sion and Lausanne, Switzerland

**Keywords:** Body awareness, Body representations, Body perceptions, Upper limb, Stroke, Sensorimotor deficits, Anosognosia, Hemiasomatognosia, Somatoparaphrenia, Disownership

## Abstract

**Background:**

Following a stroke, patients may suffer from alterations in the perception of their own body due to an acquired deficit in body representations. While such changes may impact their quality of life as well as recovery, they are not systematically assessed in clinical practice. This study aims at providing a better understanding of the rate, evolution, and impact on recovery of upper limb (UL) body perceptions (BPs) alterations following stroke. In addition, we will investigate associations among BPs alterations items, their associations with the sensorimotor functions, UL activity, damages in brain structure and connectivity.

**Methods:**

We developed a new tool named ALPQ (for Affected Limb Perception Questionnaire) to address the present study objectives. It assesses subjective alterations in the perception of the affected UL following stroke, by measuring several dimensions, namely: anosognosia for hemiplegia, anosodiaphoria for hemiplegia, hemiasomatognosia, somatoparaphrenia, personification of the affected limb, illusion of modification of physical characteristics (temperature, weight, length), illusory movements, super- or undernumerary limb, UL disconnection, misoplegia, and involuntary movement. This study combines a cross-sectional and longitudinal design. The completed data sample will include a minimum of 60 acute and 100 sub-acute stroke patients. When possible, patients are followed up to the chronic stage. Complementary evaluations are administered to assess patients’ sensorimotor and cognitive functions as well as UL activity, and brain lesions will be analysed.

**Discussion:**

This study will provide a better understanding of BPs alterations following stroke: their rate and evolution, as well as their associations with sensorimotor deficit, cognitive profile and UL activity, brain lesions and recovery. Ultimately, the results could support the personalization of rehabilitation strategy according to patients’ UL perception to maximize their recovery.

**Study registration:**

The protocol for this study has been pre-registered on the Open Science Framework on July the 7th, 2021: https://osf.io/p6v7f.

**Supplementary Information:**

The online version contains supplementary material available at 10.1186/s12883-024-03648-6.

## Background

Body representations encode “body perceptions” (BPs), that is how the body is sensed (e.g., in terms of the shape, size, weight) and experienced (i.e. sense of ownership, agency, emotional feeling towards the body parts) (e.g., [1–3]).

Body representations are not fixed, but constantly updated, in particular as a function of the bidirectional multisensory flow of information (e.g., sensorimotor, proprioceptive, visual signals) between the body and the brain [4]. Following stroke, sensorimotor deficits may alter this flow, leading to changes in the way patients perceive their body, i.e. BPs’ distortions (e.g., [5–8]). For example, stroke patients suffering from somatoparaphrenia spontaneously deny that their contralesional limb belongs to themselves, even pretending it belongs to someone else [9]. Less severe cases report altered feeling towards the contralesional limb when explicitly asked [10, 11] or alterations in the perceived dimension of the contralesional limb when experimentally tested [12, 13].

BPs’ alterations (or distortions) may impact patients’ recovery [10] and quality of life [8, 14]. However, contrary to sensorimotor functions, BPs are not systematically assessed in clinical routine practice, so that a deficit may be unnoticed unless the patients spontaneously report it.

This has led to a currently limited understanding of BPs alterations in terms of rate, patients’ profile (i.e., associated deficits and lesions), evolution and impact on the recovery of other functions. We believe that this shortcoming may be explained by a lack of tools sensitive enough to assess BPs, and simple enough to be compatible with clinical environment and routines. In the next paragraphs, we outline these current open issues and present the approach proposed in this study to address such limitations.

### Reporting of alterations in BPs after stroke

To investigate the rate of BPs alterations following stroke, previous studies focused on one specific deficit at a time, such as anosognosia for hemiplegia [15] or somatoparaphrenia [16]. However, this does not provide a broad view of the extent and diversity of BPs alterations. The overall rate of BPs deficits after stroke has only been seldom investigated. Schwoebel and Coslett [6], Raimo and co-authors [17], Razmus [18] as well as Bassolino and co-authors [13] estimated altogether from 39.5% to 81% of stroke patients with a deficit in at least one measure of BPs. This wide range is explained by the different experimental tasks used in each study, targeting different aspects of BPs (e.g., body structural description [6, 17, 18], body semantics [6, 17, 18] or metric body representations [13]). In addition, different profiles of the patients were included, varying by the disease phase (acute, sub-acute and/or chronic), the inclusion [18] or exclusion [6, 17] of bilateral lesions, the selection of specific lesion lateralisation (e.g., only right brain damage patients [11, 16]) or clinical deficits (e.g., only patients with motor deficits [13]). In particular, the choice of restrictive inclusion criteria, necessary to reach certain objectives of previous studies, limits our understanding of BPs alterations in the general stroke population.

Moreover, the above reported rates of BPs alterations were mainly estimated through task-based assessments and may not reflect the explicit experience of patients. Using a questionnaire to assess the subjective experience of patients on multiple items targeting explicit feelings towards the affected limb (e.g., I feel my arm as “foreign”, or “dead”), Bassolino et al. found that 100% of 60 chronic stroke patients with unilateral motor deficit reported at least two negative feelings towards their affected upper limb (UL) [13].

### Evolution of BPs alterations

Although BPs distortions can be long lasting (e.g., [10, 13, 19]), they are more frequently observed in the acute phase after stroke (e.g., [20]). One explanation is that some disorders, like anosognosia for hemiplegia or somatoparaphrenia, can resolve a few days (but also later) after stroke (e.g., [20]). Moreover, we suspect that after the first confrontations with therapists in earlier stages, patients may consider unnecessary or embarrassing to complain about them afterwards. Therefore, to get a comprehensive view of BPs alterations after stroke, it is crucial to study their evolution through the different stages of the disease.

Using the Bath CRPS Body Perception Disturbance Scale (BPD) [21], initially developed for Complex Regional Pain Syndrome (CRPS) patients, Serrada et al. [14] investigated the evolution of some components of BPs (affected limb ownership; awareness of its position; attention to the limb; emotional feelings toward the limb; the subjective mismatch between vision or touch of the affected limb and its perception in terms of size, weight, pressure and temperature; the desire of amputation of the affected limb; the mental representation of both affected and unaffected limbs using the unaffected limb as a comparator) in stroke patients from the acute to the chronic phase. They report that some BPs disturbances captured through the BPD scale in the acute phase improved one month after stroke, with no further improvement up to six months after stroke. While this result offers a first insight on a possible favourable evolution of some BPs alterations after stroke, the authors did not detail the results for each sub-component of the BPD scale, thus leaving interpretations related to specific BPs alterations impossible. Instead, using different assessment tasks, Crema et al. [10] showed that alterations in UL BPs (namely the underestimation of the perceived arm length as assessed through the body-landmarks localization task, and patients’ negative feelings towards their affected limb evaluated with the Affected Limb Explicit Feelings questionnaire (ALEFq)) can still be present at the chronic stage and can be ameliorated following specific interventions. However, the study included patients at the chronic stage with no information on the earlier status, preventing conclusions about the evolution of BPs alterations. The lack of other studies highlights the need to further investigate the evolution of BPs alterations assessed with stroke-specific tools along the different phases of the disease and in relation to the recovery of other functions.

### Relationship with sensorimotor deficits

Since the construction and update of BPs resides particularly (although not exclusively) in the bidirectional flow of sensorimotor signals, it is critical to quantify the presence of sensory and motor deficits and to study their relationship with BPs alterations to better understand their underlying mechanisms and their impact on patients’ recovery. Serrada et al. [14] reported that distortions in UL perception strongly correlated with motor impairment, but weakly correlated with superficial somatosensory and proprioceptive deficits. Meanwhile, Crema et al. [10] reported that, in the chronic phase, BPs distortions were associated with proprioceptive deficits. Although these studies provide some seminal notions on this topic, the relationship between BPs and sensorimotor deficits remains to be better explored by assessing them along the different phases of the disease and with respect to recovery, in patients with a broad range of sensorimotor abilities.

### Lesion correlates of BPs distortions

Finally, BPs alterations after stroke may depend either “indirectly” on brain lesions causing sensorimotor deficits, or on lesions affecting brain areas or networks processing body signals to update body representations. The study of associations between BPs distortions and brain lesions has been frequently addressed by focusing on hemispheric lateralization (e.g., [17]) or on structural lesions (via voxel lesion symptom mapping, VLSM), thus identifying brain regions associated with a specific BPs alteration (e.g., [19, 22, 23]). More recently, new approaches studying the networks have been used (e.g., [24–26]), suggesting that network’s disconnection may better explain alterations in BPs than damage of discrete cerebral areas [5]. However, these recent studies focused only on a specific neuropsychological alteration (e.g., personal neglect, body ownership or anosognosia) without allowing comparison between different BPs alterations.

### Tools to assess BPs distortions

In the literature, the existing tools to assess BPs (non-exhaustive lists can be found in Bassolino and Serino, 2022 [5], and Serrada et al., 2022 [27]) present some limitations which make them unsuitable to address the questions that are the focus of this study (see below). Indeed, previous studies propose tasks or questionnaires that are primarily limited to the exploration of a single BPs alteration (e.g., [16]), include items not specific to the stroke population [10, 14], and/or have a qualitative approach (e.g., [8, 28]). In addition, most likely due to the clinical context and/or patients’ capabilities, sometimes a less sensitive scoring system based on binary answers (e.g., the ALEF questionnaire [10]) or Likert scale (e.g., the MUNA questionnaire [11]), has been applied. See Additional File Table A1 for a summary of the above listed examples.

For these reasons, to address the present study objectives, we developed a new questionnaire to capture patients’ explicit disturbances towards their affected limb, the Affected Limb Perception Questionnaire (ALPQ) (see “Methods/design” section).

### Aim of the study

This project aims at better characterizing disturbances in the perception of the affected UL following stroke by:

(i) estimating the rate and the severity of each potential BPs distortions;
(ii) exploring the relationship of BPs alterations between each other as well as with sensorimotor deficits and UL activity;
(iii) assessing the evolution of BPs alterations and their impact on sensorimotor recovery and UL activity;
(iv) characterizing structural lesions to specific brain areas or networks disconnections associated to BPs distortions.

## Methods/design

### Study design

**Fig. 1:**
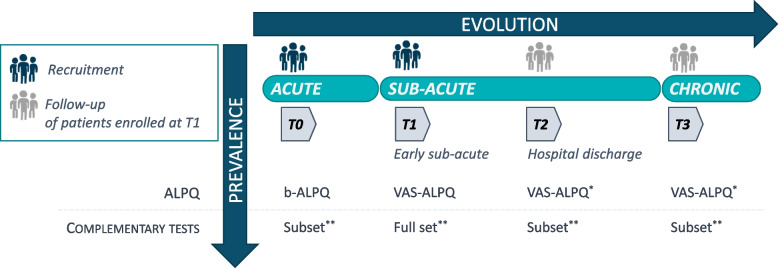
Illustration of the ALPQ study design. Patients are recruited at two timepoints: T0 (acute) and T1 (early sub-acute); Patients enrolled at T1 are invited for a follow-up visit at T2 (at their hospital discharge) and T3 (chronic phase) timepoints; (*) The VAS-ALPQ may only be performed if patient reported a positive symptom to at least one item of the ALPQ administered at the previous timepoint; (**) See Table 2 for the list of tests performed at each timepoint

This study combines a cross-sectional and a longitudinal design, summarized in Fig. 1.

Participants are recruited at different stages of the disease:

- [T0] in the acute phase of stroke (up to 14 days after stroke event) in one stroke unit (Lausanne University Hospital (CHUV), Lausanne, Switzerland)
- [T1] in the sub-acute phase of (> 14 days to 3 months after stroke) in 3 rehabilitation centers (Lausanne University Hospital (CHUV), Lausanne, Switzerland; Lavigny Hospital, Lavigny, Switzerland; Villa Beretta Rehabilitation Center, Valduce Hospital Como, Costa Masnaga, Italy).

In addition, sub-acute stroke patients seen at timepoint ‘T1’ are invited for a follow-up visit:

- [T2] before their hospital discharge (only if T2 ≥ T1 + 3 weeks)
- [T3] at the chronic phase (from 6 to 18 months following stroke event, only if T2 evaluation has been completed)

Due to clinical organisation, this follow-up is conducted systematically at one study site (Villa Beretta Rehabilitation Center), and with a limited number of patients at a second study site (Lavigny Hospital).

Patients seen at the acute phase (timepoint T0) are not followed-up at the sub-acute phase, unless they are hospitalized in one of the study rehabilitation hospitals where they are invited to participate in the sub-acute phase of the study.

A short version of the ALPQ (see b-ALPQ described hereafter) is administered at T0 to study the rate of BPs alterations in the acute phase. A complete version of the ALPQ (see VAS-ALPQ described hereafter) is administered at T1, T2 and T3 to evaluate the evolution of BPs deficits and their recovery.

Complementary evaluations to assess UL activity as well as sensorimotor and cognitive functions are performed at ± 7 days from the administration of the ALPQ in all phases (see Table 2 for the list of these tests).

In addition, brain lesion analyses will be conducted on available neuroradiological images at the acute stage (and/or early sub-acute stage) acquired for clinical purposes, whether MRI or Angio-CT scan.

### Study population / participants

Patients fulfilling the following criteria are invited to participate in the study. Note that inclusion and exclusion criteria have been defined with the aim of being representative of the general stroke population (i.e., including unilateral and bilateral lesions, with and without sensorimotor deficits) to better assess the rate of BPs alterations, while ensuring a correct administration of the ALPQ.

Inclusion criteria:

- Age ≥ 18 years old.
- Diagnosis of stroke (≤ 14 days for the acute phase, 15 days to 3 months for the sub-acute phase).
- Ability to understand and respond to instructions (as determined by the clinician). Importantly, this includes the ability of the patient to be able to look at the contralesional limb or to verbally indicate that he/she has understood that the question is not about the ipsilesional limb but the other limb.
- Sufficient understanding of verbal requests of French/Italian (all neuropsychological tests can be administered in French/Italian).
- Ability to understand the patient information sheet, and to sign the consent.
- In addition, for the VAS-ALPQ, the ability to indicate response on a vertical visual analogue scale is assessed prior administration of the ALPQ.

Exclusion criteria:

- Major neurological diseases other than stroke: neurodegenerative diseases (e.g., Parkinson’s disease, dementia) or auto-immune diseases (e.g., multiple sclerosis) or meningoencephalitis or leukopathia.
- Patients with a diagnosed epilepsy before stroke that required surgery or medications.
  Importantly, stroke-related epilepsy episodes are NOT an exclusion criteria (even if they are under medications).
- History of brain tumor.
- History of moderate or severe brain trauma (defined as Glasgow score < 13 or post-traumatic amnesia > 24h).
- Recurrence of stroke, if the last stroke event occurred within 6 months from the previous one. It is accepted only IF previous lesions occurred more than 6 months ago, wherever the lesions side(s).
- History or current psychosis (e.g., Schizophrenia) or eating disorders (in agreement with DSM 5 definitions).
- Unresolved somatosensory and/or motor deficit, unrelated to stroke, of any UL (contralesional or ipsilesional). Importantly, sensorimotor deficit related to a past stroke event are NOT an exclusion criteria.

We aim at enrolling at least 60 eligible acute patients (at T0) and 100 eligible sub-acute patients (at T1). See Additional file 1 for details about the sample size. The enrolment is ongoing at the time of submission of this manuscript.

In order to estimate the rate of BPs distortions after stroke, the screening and enrolment is done consecutively. At the acute stage, all stroke patients seen by the neuropsychological service in a stroke acute unit are screened. At the sub-acute stage, all stroke patients admitted to a hospital’s rehabilitation units are screened. The recruitment started in November 2021 and is estimated to last between 20 to 36 months. A group of at least 30 age-matched healthy controls has also been evaluated to assess possible variability in the responses given on the VAS scale [16].

### Measurements

#### The Affected Limb Perception questionnaire (ALPQ)

We have designed a structured questionnaire to assess subjective disturbances in the perception of the affected UL following stroke. We developed two versions of the questionnaire (see Additional files 2 and 3): (i) a short version (the b-ALPQ, b- for ‘binary’), based on binary yes/no answers, compatible with acute patients’ vigilance, fatigability and availability in an acute stroke unit and (ii) a standard version (VAS-ALPQ), for patients seen at later stages of the disease, which aims at providing a more sensitive and quantitative assessment of the deficits by using a continuous visual analogue scales (VAS). Other authors already shown the efficacy of VAS scales to detect and investigate body disownership after a brain lesion [16].

The questionnaire assesses a wide spectrum of BPs related alterations, listed in Table 1: it includes frequently documented alterations (e.g., anosognosia for hemiplegia, somatoparaphrenia) as well as additional less documented ones identified from our experience with stroke patients (namely UL disconnection, change in physical characteristics, illusory movements). The evaluation of additional BPs deficits (anosognosia for hemiasomatognosia, over-care for the UL, etc.) were initially included in the questionnaire, but removed in order to keep the time of administration of the questionnaire compatible with clinical practice. Since some studies suggest that pain could alter body perception [29], and that it is a common symptom after stroke [30, 31], the ALPQ also assesses pain as control item.

**Table 1:** Items included in the b-ALPQ and the VAS-ALPQ

	**b-ALPQ**	**VAS-ALPQ**
	*yes/no*	*VAS*
Fatigue		X
Sadness		X
Anxiety		X
**Most-affected limb**		
1. Pain	X	X
2. Anosognosia for hemiplegia	X	X
3. Anosodiaphoria for hemiplegia	X	X
4. Hemiasomatognosia	X	X
5. Somatoparaphrenia	X	X
a) Upper limb belonging to someone else		
b) Upper limb not being human		
6. Personification of the affected limb	X	X
7. Illusion of modification of physical characteristics	X	X
a) Temperature		
b) Weight		
c) Length		
8. Illusory movements	X	X
9. Supernumerary /Undernumerary limb	X	X
10. Upper limb disconnection	X	X
11. Misoplegia	X	X
12. Involuntary movements (lack of agency)	X	X
**Less-affected limb**		
1. Anosognosia for hemiplegia		X
2. Hemiasomatognosia		X
3. Somatoparaphrenia (Upper limb belonging to someone else)		X
**Evaluation of altitudinal neglect**		
Altitudinal neglect		X

The items are always presented in the same order, as listed in Table 1. In addition to the BPs alterations related items that are included in the b-ALPQ, the VAS-ALPQ includes three initial items to assess the general status of the patient at the moment of the administration: fatigue, sadness, and anxiety. Moreover, three BPs alterations related items regarding the less-affected UL are administered as control items, after the evaluation of the most-affected UL, as described in Table 1. Finally, altitudinal neglect (also called vertical neglect) [32–36] is evaluated at the end of the questionnaire. It is not assessed at the beginning in order to limit the risk of inducing a persistent response to the middle of the scales. A description of the altitudinal neglect assessment is available in Additional file 1.

In case of unilateral cortical lesion, the most affected UL is the one contralateral to the lesion, while in case of cerebellar lesion, the most affected UL is the ipsilesional one. In case of bilateral lesions, the limb with the highest sensorimotor deficits (based on the clinical sensorimotor evaluation) at T0 or T1 – or contralateral to the most affected hemisphere (based on the total volume of the lesion observed by the clinician on neuroradiological images) in the unlike case of no sensorimotor deficit – is considered as the most affected one.

In the acute phase, patients are asked to report their feelings in the present and past 24 h. In the sub-acute and chronic phase, the questions refer to the past 7 days.

In this study, the b-ALPQ is in French language, and the VAS-ALPQ in French and in Italian. Depending on the patient’s condition and answers to the questionnaire, the b-ALPQ is administered in 5 to 10 min and the VAS-ALPQ in 20 to 50 min.

##### The b-ALPQ

The b-ALPQ is administered verbally to the patient i.e., the questions are read by the examiner and the patient answers verbally with “yes” or “no” answers.

If the patient reports an altered BP sensation (i.e. “yes” answer), he/she is invited to further describe the characteristics of her/his altered sensation (if any), through structured questions, for the following items: somatoparaphrenia, personification of the affected limb, illusion of modification of physical characteristics, illusory movements, supernumerary/undernumerary limb, UL disconnection and involuntary movements.

##### The VAS-ALPQ

In order to allow the questionnaire to be administered to patients suffering from visual hemineglect, the VAS are presented vertically and centred on the patient’s midline, as previously described by other authors [16]. We developed an ad-hoc application to administer the VAS-ALPQ using a tablet. To run the app, we chose a Samsung Galaxy Tab A7, which has a symmetrical frontal layout and does not contain any element that could bias the attention of the patient towards the top or bottom of the screen.

**Fig. 2:**
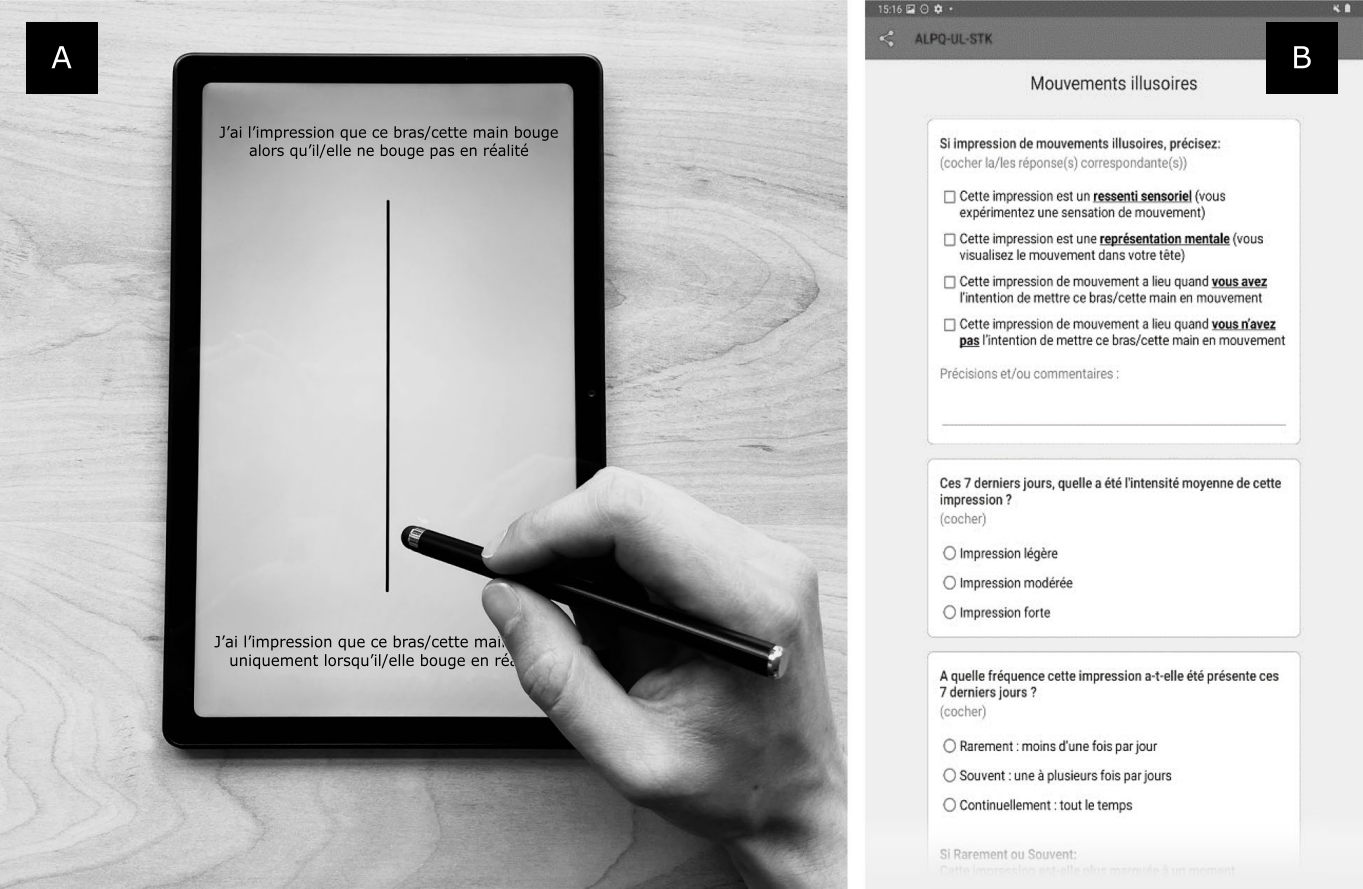
Example of administration of the VAS-ALPQ. **A** Patient answering with a stylet the VAS to evaluate *Illusory movements* in the French version of the questionnaire. At the top of the VAS: *“I feel that this arm/this hand is moving when in fact it is not”*. At the bottom: *“I feel like this arm/this hand only moves when it actually moves”*. **B** Example of complementary questions asked to patient in case s/he reports *Illusory movements*: 1) further characteristics of patient’s impression (upper box: *“If feeling of illusory movements, specify (check the corresponding answer(s)): This feeling is a sensory feeling (you experience a sensation of movement); This feeling is a mental representation (you visualize the movement in your head); This feeling of movement takes place when you intend to put this arm/this hand in motion; This feeling of movement takes place when you do not intend to put this arm/this hand in motion”*), 2) intensity (central box: *“Over the last 7 days, what has been the average intensity of this feeling? Light; Moderate; Strong”*) and 3) frequency (lower box: *“How often has this feeling been present over the last 7 days? Rarely: less than once a day; Often: one to several times a day; Continuously: all the time”*)

A sentence is presented at the top and at the bottom of the scale, and the patient is asked to indicate her/his response in relation to the two statements (see Fig. 2A). The sentence describing a BP alteration is always positioned at the top of the scale, while the opposite one (i.e. describing no BP alteration) is positioned at the bottom. The top of the scale corresponds to a score of 14 (i.e., the maximum degree of perceived alteration), while the bottom corresponds to zero (i.e., the absence of alteration). Any answer above 0 indicates the presence of an alteration, and the score reflects the degree of the reported sensation.

For each item of the VAS-ALPQ, the examiner reads the top and bottom sentences of the VAS, pointing at them with the stylet. Then the patient answers the VAS directly on the tablet, using the same stylet (see Fig. 2A). If the patient’s answer on the VAS is spatially close to the bottom (i.e. zero), the examiner asks the patient to verbalise her/his answer in order to understand whether s/he was aiming to report a mild alteration (VAS > 0 but close to zero) or no alteration.

For each item of the VAS-ALPQ, in case of an answer indicating a BP alteration (any time the value of the corresponding VAS is > 0), the examiner asks verbally the patient to rate on a 3-points scale the intensity and frequency of the reported sensation. The intensity and frequency, together with the quantitative score on the VAS, will allow to characterize the severity of the perceived sensation. The answers to these additional questions are collected directly on the application (see Fig. 2B) and may also allow to detect a misunderstanding of an ALPQ item or a contradiction in patients’ answers (e.g., if a very high frequency and intensity is reported in complementary questions, while a very low score on the VAS scale is indicated). If there is a contradiction in the answers, the examiner may clarify the response by asking the patient the same ALPQ VAS question a second time, without confronting her/him directly with the inconsistency, so as to avoid influencing her/him. These details are well specified in the instruction manual (see in the paragraph ‘Instructions’). Moreover, similarly to the b-ALPQ, additional structured questions are asked verbally by the examiner to guide the patient to further describe the characteristics of the reported sensation for the following items: somatoparaphrenia (e.g., *“Whose arm/hand is this?”*), personification of the affected limb, illusion of modification of physical characteristics, illusory movements, supernumerary/undernumerary limb, UL disconnection and involuntary movements.

Before the administration of the VAS-ALPQ, two preliminary evaluations are performed to ensure that the patient is able to answer the VAS-ALPQ and allow her/him to familiarize with the procedure: (1) the patient’s ability to perceive the vertical line and discriminate its two extremities is evaluated by asking her/him to indicate the upper and lower extremities of a vertical line and to follow the line with the stylet; (2) the patient’s understanding of the use of a VAS is verified with two examples. These examples do not concern the body and suppose that the patient answers at the upper extremity of the scale for the first example, and between the two extremities for the second example.

##### Instructions

Several key rules are followed when administering the questionnaire. For example, when asking the questions related to the UL, the examiner points at the arm/hand saying “this arm/this hand” but never touches it, nor uses the pronoun “your” (“your arm/your hand”). This prevents to give tactile stimulation on the affected side or to perform a confrontation with a potential feeling of disownership.

In order to administer the VAS-ALPQ to all patients in the same way, and to ensure the examiner is careful not to influence the patients’ responses throughout the questionnaire, an instruction manual was created (indicating, for instance, when to accept a request from the patient to change her/his answer and how to record it) and a specific training was provided to all examiners.

#### Complementary evaluations

Complementary evaluations are collected within ± 2 days to the administration of the b-ALPQ at T0, and within ± 7 days to the administration of the VAS-ALPQ at T1, T2 and T3. The following functions are assessed: a) *Somatosensation*, including superficial sensation, as well as static and dynamic proprioception; b) *UL motor function*, including force, strength, spasticity; c) *UL activity* is assessed by patient’s therapist in a 4-point Likert scale for all patients at T0, T1 and T2, as well as, at one study site (Villa Beretta Rehabilitation Center), with accelerometer bracelets on both wrists at T1 and T2; d) *Cognitive functions*, including memory, personal neglect, language, executive functions and UL apraxia; e) *Anxiety and depression*. The full set of tests are listed in Table 2.

**Table 2:** List of complementary evaluations collected at each timepoint

	**Stroke patients**				**Control subjects**
	**Acute**	**Sub-Acute**		**Chronic**	
	*T0*	*T1*	*T2*	*T3*	
**Affected Limb Perception Questionnaire**					
b-ALPQ	**X**				
VAS-ALPQ		**X**	**X**	**X**	**X**
**Somatosensory functions of the upper limb**					
*Superficial*					
Tactile detection (Em-NSA) [37]		**X**	**(X)**	**(X)**	
Sharp/Blunt discrimination (Em-NSA) [37]		**X**	**(X)**	**(X)**	
2-point discrimination test		Subgroup	Subgroup	Subgroup	**X**
*Proprioception*					
Dynamic (RASP) [38, 39]	**X** (finger only)	**X**	**(X)**	**(X)**	
Static (Thumb Localizing Test) [40]		**X**	**(X)**	**(X)**	
**Motor function of the upper limb**					
Grip strength^a^ [41]		**X**	**X**	**X**	**X**
Fugl-Meyer UL [42]		VB	VB	VB	
Short Fugl-Meyer UL [43]	**X**	**X**	**X**	**X**	
Ataxia (item from the Fugl-Meyer UL)^a^ [42]	**X**	**X**	**X**	**X**	
Modified Ashworth Scale [44]		**X**	**X**	**X**	
Motricity Index [45, 46]		VB	VB	VB	
ARAT [47]		VB	VB	VB	
Box and Blocks test [48]		VB	VB	VB	
9-Hole Peg test [49]		VB	VB	VB	
Physiotherapist’s feedback	**X**	**X**	**X**	Subgroup	
**Upper limb activity**					
Physio/Occupational therapist’s feedback	**X**	**X**	**X**	Subgroup	
Arm use (ARYS™ pro accelerometer bracelets)		VB	VB		
**Body Representations**					
Fluff test [50]		**X**	**(X)**	**(X)**	
Personal Neglect (BEN) [51]	**X**	**X**	**(X)**	**(X)**	
Bilateral Extinctions (BEN) [51]	**X**	**X**	**(X)**	**(X)**	
**Neuropsychological evaluations**					
MoCA [52]		**X**	Subgroup	Subgroup	**X**
Apples test [53, 54]		**X**	**(X)**	**(X)**	
HADS [55]		**X**	**X**	**X**	
TICSf-12 (for French speaking sites) [56]		**X**	Subgroup	Subgroup	
AAT (for Italian speaking site) [57]		**X**	Subgroup	Subgroup	
Trail Making Test [58] /Color Trail Test [59]		**X**	**X**	Subgroup	**X**
RBMT faces or objects [60]		**X**	**(X)**	**(X)**	
Digit Span Forward and Backward [61–64]		**X**	Subgroup	Subgroup	**X**
Mahieux-Laurent’s test for apraxia [65]		**X**	**(X)**	**(X)**	
Neuropsychologist/Speech therapist’s feedback		**X**	**X**	Subgroup	

T0, T1, T2, T3 = timepoint of evaluation of stroke patients

X = for all patients/subjects; (X) = only for patients who did not have the maximum score at the previous timepoint

VB = Performed only at one rehabilitation site (Villa Beretta Rehabilitation Center)

Subgroup = Data collected only if evaluation is performed in routine practice or if time allows

*Abbreviations:* *Em-NSA* Erasmus modified Nottingham Sensory Assessment, *RASP* Rivermead Assessment of Somatosensory Performance, *ARAT* Action Research Arm Test, *MoCA* Montreal Cognitive Assessment, *HADS* Hospital Anxiety and Depression Scale, *TICSf-12* short version of the “Test Informatisé de Compréhension Syntaxique en français”, *AAT* Aachener Aphasie Test, *RBMT* Rivermead Behavioural Memory Test

^a^when feasible by the patient

When applicable, the less-affected limb is always assessed before the most-affected limb.

The procedure for the administration of each complementary evaluations as well as the outcome measures are further described in Additional file 1.

## Statistical analysis

ALPQ items for which the patients showed to have not understand the question or contradicted him/herself when verbally formulating her/his answer will be reported and excluded from the analysis.

### Main statistical analyses

We aim at performing the main following analyses:

(i) The *rate* of BPs alterations following stroke (i.e. binary scores) as assessed by the ALPQ will be determined at the acute (T0) and at the early sub-acute (T1) stages as the percentage of patients presenting the deficit on the total number of patients who answered the question.
(ii) The *evolution* of BPs alterations across the various stages of the disease (from the early sub-acute phase to the chronic phase) will be studied by comparing within-subjects data between T1, T2 and T3 (e.g., through chi-square or linear mixed models or ANOVA or equivalent non-parametric analyses, in case of non-normal data distribution).
(iii) *Associations* among BPs alterations items will be assessed (e.g., through multiple correlations, cluster analyses, or principal components analyses) to identify clusters regrouping multiple items likely referring to similar BPs components.
(iv) *The relation* between ALPQ scores and the results from complementary measures will be explored (e.g., through multiple regression models or linear mixed models).

When applicable, the above analyses (as well as lesions analyses described in the next paragraph) will be performed on:

- binary scores (presence/absence of BPs alterations for b-ALPQ and VAS-ALPQ, see paragraph VAS-ALPQ in the “Methods/design” section) of specific questionnaire’s items,
- continuous scores (for the VAS-ALPQ) of specific questionnaire’s items,
- weighted binary scores on the intensity or frequency (for the VAS-ALPQ) of specific questionnaire’s items.

For each above type of score: the score of the cluster of similar items (see above point iii), or of all the items in the questionnaire, can be integrated to obtain a cumulative score. Thus, we will distinguish the *binary cumulative score*, the *continuous cumulative score*, and the *weighted cumulative score*.

While the continuous and weighted scores will include information about the intensity and frequency of the altered feeling, the binary score will be a general indicator of the presence of altered perceptions.

Analysis “i” and “ii” above will be run by adding covariates of interest in terms of patients’ lesions (e.g., side of lesion) or clinical scores (e.g., somatosensory and motor scores, see paragraph “Complementary evaluations” in the “Methods/design” sections).

### Lesion analyses

The scores detailed in the previous paragraph will be used as behavioural indexes in a series of lesion analyses aiming to understand the neural correlates of BPs alterations.

Lesions obtained for clinical purposes will be delineated to run voxel-based Lesion-Symptom Mapping analyses [65] on the specific behavioural indexes.

To reveal the correlates of BPs disturbances at the network level, further analyses based on structural or functional connections/disconnections using voxel lesion network mapping (e.g., [66]) and disconnection analysis (e.g., [26, 67, 68]) will be performed on the same behavioural index.

Lesion volume and clinical complementary measures (e.g., sensorimotor deficits, see paragraph “Complementary evaluations” in the “Methods/design” section) will be considered in all the analyses.

## Discussion

To date, it is well accepted that stroke patients may suffer from a distorted perception of their body (e.g., [6, 18]). However, these deficits are not assessed systematically. This may be explained by a lack of tools to assess BPs alterations adapted to the clinical practice, as well as by a still limited understanding of the rate, evolution, and impact of BPs alterations on the recovery of other functions.

The design of the current study will allow us to investigate four important points to better understand BPs alterations following stroke.

First, this study will provide an estimation of the *rate* of BPs alterations at the acute and sub-acute stage of the disease for the general stroke population. Traditionally, due to putative hemispheric specialization, BPs deficits are studied in right brain damaged patients (e.g., [11]), whereas other studies selected patients for the presence of sensorimotor impairments (e.g., [13]). Although, these subpopulations of stroke patients may indeed more likely suffer from BPs alterations, some studies show that BPs alterations are not limited to these cases (e.g., [17]). The eligibility criteria of the present study have been determined to enhance our understanding of BPs alterations in the general stroke population, without a priori selection in terms of patients’ lesion localisation or sensorimotor deficit, while guaranteeing feasibility of patients’ participation. Despite these broad eligibility criteria, we can still expect unbalanced groups between right and left brain hemispheres injuries, as patients in the latter group are more likely to suffer from aphasia. If aphasia limits their understanding, they are not be able to participate in the study. Although some left-brain damage patients are excluded for this reason, not limiting our observations to specific lesion laterality, nor sensorimotor impairment, represents an important opportunity to assess possible associations between different rate or severity in BPs alterations, hemispheric lateralization and sensorimotor deficits (see next point). This will also allow us to describe the complete clinical profile of patients showing BPs distortions. On another hand, it is worth noting that the reported feelings may fluctuate in time [69]. To get a comprehensive view of BPs alterations phenomenon, it was therefore important to enquire the patients about their feeling within a certain range of time rather than at the exact moment of questionnaire administration. We set this range at 24 h for the acute patients, and 7 days for the later stages of the disease. This time window was decided after consulting practicing neuropsychologists, considering the stage of the disease and patients’ memory capacity. For this reason, patients who present severe memory deficits as per their standard clinical evaluation are considered unable to respond to the instructions and are therefore not eligible. Despite this precaution, we cannot rule out the possibility that a patient reports a feeling that occur before the time range, or at the opposite misses to report a feeling due to its absence at the time of administration of the questionnaire. Although this limitation must be considered, it is intrinsic to any instrument based on subjective evaluations.

Second, the administration of a battery of complementary evaluations assessing sensorimotor and cognitive functions as well as UL activity will allow us to further characterize *associations of BPs alterations with sensorimotor deficit, cognitive profile and UL activity*. Since patients’ availability and fatigue were a limited factor, the selection of complementary tests was done considering the sensitivity of the test, its time of administration, availability of the tests and trained personnel in the involved clinical centres, as well as the possibility to have parallel linguistic versions in the involved countries (Italy and Switzerland). Ultimately, the relationship between BPs alterations and the above-mentioned functions will be explored within the limit of each complementary test.

Third, with the longitudinal design of this study, we will provide insights into the *evolution of BPs alterations up to the chronic phase of the disease*, also in relation with arm activity and recovery of sensorimotor functions. While we expect to have a higher rate at the early stages, studies have shown that some alterations may be persistent (e.g., [10, 16]). It is worth noticing that because of the patients’ clinical flow, only a minimal number of patients seen at the acute stage (T0) are transferred in one of the sub-acute study site. This implies that that most of the data obtained at the follow up in the chronic stage (T3) will have a correspondence in the sub-acute stage (T1), but not necessarily in the acute phase. Therefore, the evolution of BPs alterations will be monitored between the early sub-acute stage (T1), the end of the treatment (T2) and the chronic stage (T3).

Finally, the lesion analysis will provide insight into the anatomical basis of BPs alterations, but also their underlying network.

To tackle the present study objectives, we developed a new questionnaire, the ALPQ (Affected Limb Perception Questionnaire) with four crucial characteristics.

First, the broad range of BPs distortions covered by the ALPQ will allow us to assess their rate at the acute and sub-acute stages of a broad spectrum of BPs alterations after stroke and to track their evolution.

Second, the ALPQ has been designed to be adapted to the clinical context. Both versions of the ALPQ can be used with a wide range of patients, even those suffering from aphasia who have preserved understanding, since the use of verbal answers (beyond “yes/no” for b-ALPQ and for the complementary questions of both ALPQ versions) are not mandatory (for the VAS-ALPQ a booklet to point at the corresponding answer to complementary questions was also created). The use of the tablet allows an efficient way to administer the questionnaire and collect the data. The ALPQ administration manual and a specific training to all examiners ensure reproducibility in the procedure. To allow assessing BPs in the acute phase and in stroke units, where time for evaluation and patients’ attentional resources are limited, we had to develop a short, binary version of the ALPQ (b-ALPQ). Although the b-ALPQ would be likely less sensitive than the VAS-ALPQ based on continuous scale (see next point), this is compatible with the clinical needs and was therefore a necessary compromise. On the other hand, the b-ALPQ (which is aimed to be administered verbally) can also be used with patients suffering from major visual deficits and who may therefore not be able to answer the VAS-ALPQ.

Third, the VAS-ALPQ exploits a quantitative approach based on visual analogue scales, recently proposed to be more sensitive to capture BPs alterations [16].

Finally, the items have been defined to balance negative with positive wording, to avoid biasing patients’ responses [10]. This will allow the VAS-ALPQ to be robust in assessing the severity of distortions and monitoring their potential evolution since the sub-acute stage, as well as to conduct powerful statistical analyses on the underlying associations with sensorimotor deficits and brain lesions. These last two characteristics, quantitative approach and the balance between negative and positive wording, are however specific to the VAS-ALPQ therefore limiting the sensitivity of the evaluation of BPs alterations at the acute stage (where the b-ALPQ is used to comply with the clinical context and patients’ abilities, as described above) and of their evolution between the acute stage and the later stages of the disease.

The ALPQ is an experimental tool created to address the present study objectives. It was created by integrating neuroscientific knowledge in the field of body representations and clinical experts in stroke assessment and treatment, although no formal validation procedure (e.g., Delphi-consensus) was fully implemented. For correct administration of the ALPQ, a short training is strongly recommended and can be obtained by contacting the corresponding authors. On the other hand, while this study focuses on stroke patients, the ALPQ can be adapted and used to assess BPs in other populations with sensorimotor disorders or diseases that can lead to alterations in BPs. Adapted versions of the questionnaire to other populations (such as patients with CRPS and multiple sclerosis), targeting lower limbs, and in other languages are in preparation.

## Conclusion

The present study aims at describing a broad picture of BPs alterations after stroke in the different phases of the disease and in relation to sensorimotor deficits, UL activity, brain lesions as well as recovery. This knowledge would potentially drive the development of new rehabilitative targeted and personalized interventions taking into account BPs disturbances to improve patients’ functional outcome [8, 70].

### Supplementary Information

**Supplementary Material 1.**

**Supplementary Material 2.**

**Supplementary Material 3.**

## Data Availability

No datasets were generated or analysed during the current study.

## Abbreviations

ALPQ: Affected Limb Perception Questionnaire
b-ALPQ: Binary ALPQ
VAS-ALPQ: Visual Analog Scale ALPQ
ALEFq: Affected Limb Explicit Feeling questionnaire
BPD scale: Body Perception Disturbance scale
BPs: Body Perceptions
CRPS: Complex Regional Pain Syndrome
MUNA: Motor UNawareness Assessment
UL: Upper Limb
VAS: Visual Analog Scale
