# Supplementary material for: Evaluation of upper limb perception after stroke with the new Affected Limb Perception Questionnaire (ALPQ): a study protocol
Source: BMC Neurol. 2024 Jun 11;24:196. doi: 10.1186/s12883-024-03648-6 (PMC11165777; doi:10.1186/s12883-024-03648-6)
Supplement: Supplementary file 1 — Supplementary Material 1. [file 12883_2024_3648_MOESM1_ESM.pdf]

ADDITIONAL MATERIAL
Additional file 1

This document provides further information on (A) currently existing questionnaires and interviews, (B) the sample size, and (C) the altitudinal neglect evaluation and on the complementary tests mentioned in the METHODS section of the manuscript.

Contents

A. TOOLS TO ASSESS BPs DISTORTIONS 3
B. SAMPLE SIZE 4
C. ALTITUDINAL NEGLECT AND COMPLEMENTARY EVALUATIONS 5
1 Altitudinal Neglect 5
2 Somatosensory functions of the upper limb 5
2.1 Em-NSA (Tactile sensation and Sharp-Blunt discrimination) 5
2.2 Two-Point Discrimination Task (2PDT) 5
2.3 Dynamic proprioception (Rivermead Assessment of Somatosensory Performance, RASP) 6
2.4 Static proprioception (Thumb Localizing Test, TLT) 6
3 Motor function of the upper limb 6
3.1 Grip strength 6
3.2 Fugl-Meyer Assessment Upper Limb (FMA-UL) 6
3.3 Short Fugl-Meyer Assessment Upper Limb (Short FMA-UL) and Ataxia 7
3.4 Modified Ashworth Scale (MAS) 7
3.5 Motricity Index (MI) 7
3.6 Action Research Arm Test (ARAT) 7
3.7 Box and blocks (BBT) 7
3.8 Nine-hole peg test (9HPT) 7
3.9 Physiotherapist's feedback on upper limb motor function 7
4 Upper-limb activity 7
4.1 Physiotherapist/Occupational therapists's feedback 7
4.2 Spontaneous arm use (ARYS^{TM} pro accelerometer bracelets) 8
5 Body Representations 8
5.1 FLUFF test 8
5.2 BEN (sub-items Body Neglect and Bilateral Extinction) 8

6 Neuropsychological evaluations 8
6.1 Montreal Cognitive Assessment (MoCA) 8
6.2 Apples Cancellation test 8
6.3 Hospital Anxiety and Depression Scale (HADS) 9
6.4 Test of Syntactic Comprehension (TICSf-12) 9
6.5 Aachener Aphasie Test and Test of Syntactic Comprehension (AAT) 9
6.6 Trail Making Test (TMT) / Color Trail Test (CTT) 9
6.7 Rivermead Behavioural Memory Test (RBMT) 9
6.8 Digit Span Forward and Backward 9
6.9 Mahieux-Laurent’s test for apraxia 10
6.10 Neuropsychologist/Speech therapist’s feedback 10
7 Bibliography 10

A. TOOLS TO ASSESS BPs DISTORTIONS

Table A1. Examples of existing questionnaires and interviews to assess BPs alterations in stroke patients

**Paper**	**Tool name**	**Evaluated BPs (number of items)**	**Designed for stroke patients?**	**Stroke stage of the population assessed in this study *(sample)***	**Scale: Binary, Likert, Continuous, or n/a (qualitative)**
Crema et al., 2022 [1]<br><br>Bassolino et al., 2022 [2]	Affected Limb Explicit Feelings questionnaire (ALEFq)	Explicit disturbances towards the affected limb (10 items)	No<br>*It is a combination of two questionnaires developed for CRPS patients: (1) the Feeling of foreignness questionnaire* [3] *and (2) the Neurobehavioral Questionnaire* [4]	Chronic *(N=60)*	Binary
Serrada et al., 2021 [5]	Bath CRPS Body Perception Disturbance Scale (BPD)<br>(from Lewis and McCabe[6])	Explicit disturbances towards the affected limb (10 items)	No<br>*Initially developed for CRPS patients*	Follow-up study covering acute *(N=89)*, sub-acute and chronic stages	Binary (4-items)<br>11-points Likert scale (5 items)<br>3-points Likert scale (1 item)
Moro et al., 2021 [7]	The Motor Unawareness Assessment (MUNA)	Anosognosia for Hemiplegia (40 items)	Yes	Lesion-test interval range: 1-222 days (mean 41.81 ±7.5 days) *(N=131)*	3-points Likert scale
Ronchi et al., 2020 [8]	VAS scale	Disownership of body parts (1 item)	Yes	Acute, sub-acute and chronic *(N=32)*	Continuous
Stott et al., 2021 [9]	Semi-structured interview	Explicit disturbances towards the body	Yes	Chronic *(N=16)*	n/a (qualitative)

CRPS = Complex Regional Pain Syndrome; n/a = not applicable.

B. SAMPLE SIZE

This study aims at enrolling minimum 60 acute patients (at T0) and minimum 100 sub-acute patients (at T1).

These numbers have been chosen based on (1) the sample size of other studies in the literature which estimated the prevalence of body representation alterations following stroke (between 50 in Razmus et al. [10] to 131 patients in Moro et al. [7]), and (2) recruitment capacities at the study sites in the planned time frame.

Given that the ALPQ is a new tool, no previous data are available (with a similar objective and method involving a population of patients with acute or sub-acute stroke). Therefore, only a rough estimation of sample size is possible by referring to data collected with a different, yet related, questionnaire (ALEFq), with a different aim and patients population (chronic stroke patients) [1].

This analysis recommends N=63 to detect modifications in BPs alterations after treatment i.e. T2-T1 (Wilcoxon signed-rank test (matched pairs), two-tails, Cohen's dz = -0.37, α error probability =0.05, and power (1-β error probability) =0.80, GPower version 3.1.9.4).

On the other hand, to do more complex analysis such as Voxel Lesion Symptom Mapping, a larger sample size is preferred as per Lorca-Puls DL et al. (Neuropsychologia, 2018) [11]. Therefore, we aim at enrolling a minimum of 100 sub-acute stroke patients at T1.

C. ALTITUDINAL NEGLECT AND COMPLEMENTARY EVALUATIONS

1 Altitudinal Neglect

Altitudinal neglect (also called vertical neglect) is evaluated by using the same tablet than the one used for the VAS-ALPQ. It is administered immediately at the end of the administration of the VAS-ALPQ.

We adapted the test of horizontal lines bisection from Azouvi et al. [12] as described below. The tablet is positioned so that the vertical line is centred on the patient's midline. The following instruction is given to patients: “Can you please cut this line down the middle so that it is divided, as accurately as possible, into two equal parts?”.

Four vertical lines, centered on the tablet’s screen, are presented to the patient, in the same order: (1) line of 14cm, (2) line of 7 cm, (3) line of 7cm, (4) line of 14cm.

The instructions are the same for the 4 vertical line bisections.

2 Somatosensory functions of the upper limb

2.1 Em-NSA (Tactile sensation and Sharp-Blunt discrimination)

Superficial somatosensory function is evaluated through the following two sub-items of the Em-NSA: Tactile Sensation and Sharp/Blunt discrimination test [13].

The *Tactile Sensation* subtest includes light touch, pressure and pinprick stimulation to be applied on the skin of the patient’s fingers, hand, forearm and arm. The patient indicates whether he/she felt the stimulus, while blindfolded.

In the *Sharp/Blunt discrimination* subtest, the skin is stimulated on the same areas tested at the Tactile Sensation subtest, with either the experimenter’s finger or a toothpick. The patient describes the test sensation as sharp or blunt, while blindfolded.

For each *Tactile Sensation* and *Sharp/Blunt discrimination* subtest, the less affected upper limb is tested first and then the most affected upper limb. The experimenter records the score for each segment and each tactile stimulation type as described by Stolk-Hornsveld et al. [13].

2.2 Two-Point Discrimination Task (2PDT)

The 2PDT is administered to evaluate tactile perception threshold of the upper limbs [14]. The procedure consists in a double staircase, both ascending and descending, adapted from previous studies [14, 15].

A digital calliper is used to apply two points pressure on patient’s glabrous skin, while blindfolded. The assessment is performed at two locations: the middle of the forearm and the thenar eminence. The distance between the two points (in millimetres) is computed using an adaptive method and ranges from a minimum of 20 mm (on the thenar eminence) to a maximum of 110 mm (on the forearm). Each distance perceived as two points is re-evaluated for a total of up to 3 consecutive repetitions, while each distance perceived as one point is not repeated.

The general rule for calculating the distances to be tested depends on the accuracy of patient's perception:

- If two points are correctly discriminated for three consecutive repetitions:

    - This distance is marked as “fully correct” distance
    - The next distance to be tested corresponds to half of the distance just tested;
- If two points are not correctly discriminated:
    - the next distance to be tested is averaged between the last “fully correct distance” and the last distance tested (incorrectly perceived as one point in one of the three repetition).

An Excel file with these rules is used to make the procedure more automated and to avoid calculation errors. The acquired parameters are the distance between the two points pressure, expressed in mm and patient's verbal response (one or two).

The less affected upper limb is tested first and then the most affected upper limb. The body part (forearm or thenar eminence) tested first is randomized between patients.

For each upper limb (less and most affected) and each skin location (forearm and thenar eminence), the outcome is the individual threshold calculated as the mean between the two lasts “fully correct” distances.

2.3 Dynamic proprioception (Rivermead Assessment of Somatosensory Performance, RASP)

Dynamic proprioception is assessed through the subtest *Joint Movement and Movement Direction Discrimination* of the RASP [16, 17]. The joints tested are index (or thumb), wrist and elbow, first on the less affected side and then on the most affected one. Each joint is moved up and down; the patient is asked to indicate whether he/she perceived passive movements and their directions, while blindfolded.

For each movement applied, the experimenter assigns a score of 0 if the movement is not perceived ; 1 if it is perceived but the direction of the movement (up/down) is not detected ; 2 if the direction of the moment is detected.

2.4 Static proprioception (Thumb Localizing Test, TLT)

Static proprioception is assessed through the TLT [18]. Patient’s affected arm is passively moved in three positions while blindfolded, and for each position the patient is asked to pinch the tip of the thumb with the fingers of the less affected side. The same procedure is performed first with vision occluded and then with vision restored (to exclude imprecision due to ataxic movement).

For each position, the experimenter assigns a score based on the accuracy of the reaching movement. Scores range from 0 (normal) to 3 (severe impairment) as described by Otaka et al. [18] .

3 Motor function of the upper limb

3.1 Grip strength

Grip strength is assessed with a hand hydraulic Dynamometer (brand: Jamar or Lafayette) [19]. The patient is asked to use maximum force when gripping the handle of the dynamometer, while keeping the elbow flexed at 90° and the shoulder at 0°. Three consecutive measures are performed for the less affected upper limb first, then for the most affected upper limb. The average of those three measures is then calculated.

3.2 Fugl-Meyer Assessment Upper Limb (FMA-UL)

The FMA-UL [20] is administered to evaluate upper limb impairments. The scale targets motor functioning of the shoulder, elbow, forearm, wrist and hand, balance, sensation and joint functioning

(range of motion and pain). A score from 0 (cannot perform) to 2 (full perform) is assigned for each movement performed.

3.3 Short Fugl-Meyer Assessment Upper Limb (Short FMA-UL) and Ataxia

The Short FMA-UL [21] is used to evaluate motor function of both upper-limbs. This version includes the following items: flexor synergy for shoulder elevation, extensor synergy for elbow extension, pronation and supination, shoulder flexion, repeated dorsiflexion and volar flexion and thumb adduction (grasp). In addition, when possible, ataxia is assessed, via the item Coordination/Speed of the Fugl-Meyer [20].

3.4 Modified Ashworth Scale (MAS)

The MAS is used to evaluate upper limb muscle spasticity. As described by Bohannon and Smith [22], it is performed by extending patient's limb first from a position of maximal possible flexion to maximal possible extension (the point at which the first soft resistance is met). Afterwards, the scale is assessed while moving from extension to flexion. The less affected upper limb is tested first, then the most affected one. For each muscle tested (elbow extensor and flexor, wrist flexor, finger flexor) a score between 0 (no increase in in muscle tone) and 4 (affected parts rigid in flexion or extension) is assigned.

3.5 Motricity Index (MI)

The MI [23, 24] is used as a measure of upper limb motor impairment. Three movements are evaluated on a 6-points scale, namely pinch grip, elbow flexion and shoulder abduction.

3.6 Action Research Arm Test (ARAT)

The ARAT [25] is administered to assess the upper limb performance including coordination, dexterity and functioning. A score from 0 (no movement) to 3 (normal movement) is assigned to each task performed (grasp, grip, pinch and gross movements).

3.7 Box and blocks (BBT)

The BBT assesses unilateral gross manual dexterity [26]. The patient is asked to move the maximum number of wooden blocks, one by one, from one side of the box to the other. The time provided to complete the task is 60s and the total number of blocks moved is registered.

3.8 Nine-hole peg test (9HPT)

Finger dexterity is evaluated through the 9HPT [27]. The patient is asked first to move the pegs from the box to the holes in the board, one by one and as quickly as possible. Then, to remove them, one by one, and to place them back into the box. The time required to complete the task is recorded.

3.9 Physiotherapist's feedback on upper limb motor function

Upper limb motor function is also evaluated by physiotherapists based on clinical observation of patients. Physiotherapists rate the motor function of the patients' most-affected upper limb on a 4-point scale: (1) normal (no deficit), (2) mild hemiparesis (suboptimal mobility), (3) moderate to severe hemiparesis, (4) complete hemiplegia. In addition, the therapists indicate whether the muscle tone of the patients' most-affected upper limb is normal (normal tone), spastic, flaccid or hypertonic.

4 Upper-limb activity

4.1 Physiotherapist/Occupational therapists's feedback

Physiotherapists or occupational therapists are asked to rate the functional use (i.e. use of the upper limb in activities of daily living, ADL) of the most affected upper limb based on patients' clinical observation, using a 4-point scale: (1) normal (no deficit), (2) sub-optimal (functional UL used in ADL

but sub-optimally), (3) partial (UL with partial use in ADL), (4) Non-functional UL (non-functional UL - no use in ADLs).

4.2 Spontaneous arm use (ARYS^{TM} pro accelerometer bracelets)

The spontaneous use of both the less and most affected upper limbs in daily living is assessed in a sub-group of patients by using ARYS^{TM} pro accelerometer bracelets by Tyromotion. These bracelets are equipped with an accelerometer that measure all arm activities, capturing eventual asymmetries in the functional use of the most affected and less affected upper limb. Patients in this sub-group are asked to wear one bracelet on each wrist for 48h and to continue their ordinary life at the hospital, avoiding the removal of the devices except during bathing. Bathing times and physiotherapy sessions are tracked.

5 Body Representations

5.1 FLUFF test

Body neglect is evaluated via the FLUFF test [28, 29].

For the administration of this test, the patient is blindfolded while the experimenter applies some targets (felt pads) on her/his body (on clothes or on a surgical gown). The patient is then asked to explore the body using the less affected upper limb in order to find as many targets as possible. To prevent patient from bending, we adapted the original protocol [28] by excluding 6 targets for the lower legs (below the knees), therefore the total number of targets applied is 18. There is no time limitation and the total number of targets removed is recorded as well as the location on the body of the targets left.

5.2 BEN (sub-items Body Neglect and Bilateral Extinction)

The sub-item *Test of body neglect* of the BEN (Batterie d'Evaluation de la Negligence unilatérale du Geren) [12] includes the evaluation of spontaneous gaze and head orientation, as well as hemibody neglect.

The sub-item *Bilateral extinction* assesses bilateral visual, auditory and tactile (on the forearm and on the hand) extinction.

Both sub-items are administered following the procedure described in the BEN protocol [12].

6 Neuropsychological evaluations

Cognitive functions, anxiety and depression are evaluated via the set of neuropsychological and psychological tests described below. These are standard tests used in research and/or used in routine practice.

6.1 Montreal Cognitive Assessment (MoCA)

The MoCA is used to evaluate multiple cognitive domains (visuo-spatial and executive functions, naming, memory, attention, language, abstraction, and orientation) [30]. Parallel versions of this test are administered at each study timepoint to decrease possible learning effects.

6.2 Apples Cancellation test

The Apples Cancellation Test is administered to evaluate both egocentric and allocentric forms of visual hemineglect [31, 32]. The patient has to cross all full apples in a A4 page. The experimenter records

the time of execution and counts the total number of full apples crossed, the number of false positives and the asymmetry index for both true and false positives.

6.3 Hospital Anxiety and Depression Scale (HADS)

The HADS [33] is a screening tool administered to determine the level of anxiety and depression experienced in the hospital setting.

6.4 Test of Syntactic Comprehension (TICSf-12)

The TICSf-12 [34] is used to evaluate oral and reading understanding in the French-speaking sites.

The TICSf-12 consists of one list of sentences read verbally by the experimenter, and one list of sentences read by the patient. For each list, the patient is asked to choose, among four options, the figure matching the sentence heard/read. The total score (/12) is recorded.

6.5 Aachener Aphasie Test and Test of Syntactic Comprehension (AAT)

The listening and reading comprehension subtests of the AAT [35] are used to evaluate oral and reading understanding in the Italian site. In the listening subtest, the patient is asked to choose, among four options, the figure representing the object or the sentence read by the experimenter while in the reading subtest the patient has first to read a word or a phrase, and then to indicate the most representative figure. A score ranging from 0 to 3 is assigned according to the accuracy of patient's responses.

6.6 Trail Making Test (TMT) / Color Trail Test (CTT)

The TMT part A and B [36] (respectively the CTT part 1 and 2) are administered for the assessment of attention (part A or 1) and executive functions (part B or 2).

The TMT part A requires the patient to connect circles in ascending numerical order. The TMT part B requires the patient to connect circles in ascending order, alternating between numbers and letters (cognitive set-shifting ability). The CTT [37] is administered in the French-speaking sites to non-native French patients or to patients with language disorders or to patients with a small number of years of education. In the CCT Part 1, patients need to connect circles in ascending numerical order, while in Part 2, patients have to connect circles in ascending order, alternating between colours (pink and yellow) and numbers.

The time of execution and the number of errors is recorded by the experimenter.

6.7 Rivermead Behavioural Memory Test (RBMT)

The subtest *Picture Recognition* or *Face Recognition* of the RMBT 3 [38] is administered to evaluate memory (delayed recall). The patient is shown a set of pictures (Picture Recognition subtest) / faces (Face Recognition subtest), and then is asked to recognize them among a set of pictures/faces at a later time. Patients' responses to each item (yes/no) are recorded and the total number of false positives is subtracted to the total number of true recognition (true positives) to compute the final score.

6.8 Digit Span Forward and Backward

The Digit Span Forward is administered to evaluate the short-term memory and the Digit Span Backward is administered to evaluate the working memory. Patients are presented with a random series of digits, and are asked to repeat them in either the same order (forward span) or in the reverse order (backward span). The Swiss sites use the subtests from the WAIS (version IV [39] for patients up to 79 years old, and version III [40] for older patients). The Italian site use the subtests from Monaco et al. [41, 42].

6.9 Mahieux-Laurent's test for apraxia

Upper limb apraxia is evaluated with the test from Mahieux-Laurent et al. [43] which includes three subtests: symbolic gestures (5 gestures), pantomimes (5 gestures) and imitation of meaningless gestures (8 gestures). The experimenter assigns one point to each gesture correctly performed.

6.10 Neuropsychologist/Speech therapist's feedback

Based on clinical observation, the neuropsychology and/or speech therapists are asked to rate the level of aphasia of production on a 4-points scale : (1) No difficulty (Transmits all messages independently), (2) Mild difficulties (Sometimes needs help from clinician to facilitate transmission of messages (e.g. anomia)), (3) Moderate difficulty (Clinician often needs to convey messages; the listener has difficulty understanding messages), (4) Severe difficulties (Does not transmit messages, even with the help of the clinician).

In addition, the neuropsychology and/or speech therapists are asked to rate the level of visual hemineglect on a 6-point scale: (0) no visual neglect, (1) mild visual neglect (mainly directional bias), (1.5) mild to moderate visual neglect, (2) moderate visual neglect, (2.5) moderate to severe visual neglect, (3) severe visual neglect.

Finally, the therapist indicates whether any body-related disorder has been clinically identified including prosopagnosia, heterotopagnosia, autotopagnosia or other.

7 Bibliography

1. Crema A, Bassolino M, Guanziroli E, Colombo M, Blanke O, Serino A, et al. Neuromuscular electrical stimulation restores upper limb sensory-motor functions and body representations in chronic stroke survivors. Med. 2022;3:58-74.e10.

2. Bassolino M, Franza M, Guanziroli E, Sorrentino G, Canzoneri E, Colombo M, et al. Body and peripersonal space representations in chronic stroke patients with upper limb motor deficits. Brain Commun. 2022;4:1–15.

3. Förderreuther S, Sailer U, Straube A. Impaired self-perception of the hand in complex regional pain syndrome (CRPS). Pain. 2004;110:756–61.

4. Galer BS, Jensen M. Neglect-like symptoms in complex regional pain syndrome: Results of a self-administered survey. J Pain Symptom Manage. 1999;18:213–7.

5. Serrada I, Hordacre B, Hillier S. Recovery of Body Awareness After Stroke: An Observational Study. Front Neurol. 2021;12 November:1–9.

6. Lewis J, McCabe C. Body Perception Disturbance (BPD) in CRPS. Pract Pain Manag. 2010;10.

7. Moro V, Besharati S, Scandola M, Bertagnoli S, Gobbetto V, Ponzo S, et al. The Motor Unawareness Assessment (MUNA): A new tool for the assessment of Anosognosia for hemiplegia. J Clin Exp Neuropsychol. 2021;43:91–104.

8. Ronchi R, Bassolino M, Viceic D, Bellmann A, Vuadens P, Blanke O, et al. Disownership of body parts as revealed by a visual scale evaluation. An observational study. Neuropsychologia. 2020;138 December 2019:107337.

9. Stott H, Cramp M, McClean S, Turton A. 'Somebody stuck me in a bag of sand': Lived experiences of the altered and uncomfortable body after stroke. Clin Rehabil. 2021;35:1348–59.

10. Razmus M. Body representation in patients after vascular brain injuries. Cogn Process. 18:359–73.

11. Lorca-Puls DL, Gajardo-Vidal A, White J, Seghier ML, Leff AP, Green DW, et al. The impact of sample size on the reproducibility of voxel-based lesion-deficit mappings. Neuropsychologia. 2018;115:101–11.

12. Azouvi P, Bartolomeo P, Beis JM, Perennou D, Pradat-Diehl P, Rousseaux M. A battery of tests for the quantitative assessment of unilateral neglect. Restor Neurol Neurosci. 2006;24:273–85.

13. Stolk-Hornsveld F, Crow JL, Hendriks EP, van der Baan R, Harmeling-van der Wel BC. The Erasmus MC modifications to the (revised) Nottingham Sensory Assessment: A reliable somatosensory assessment measure for patients with intracranial disorders. Clin Rehabil. 2006;20:160–72.

14. Mancini F, Bauleo A, Cole J, Lui F, Porro CA, Haggard P, et al. Whole-body mapping of spatial acuity for pain and touch. Ann Neurol. 2014;75:917–24.

15. Canzoneri E, Ubaldi S, Rastelli V, Finisguerra A, Bassolino M, Serino A. Tool-use reshapes the boundaries of body and peripersonal space representations. Exp Brain Res. 2013;228:25–42.

16. Winward CE. The Rivermead Assessment of Somatosensory Performance (RASP) - Operator Manual. 2012.

17. Winward CE, Halligan PW, Wade DT. The Rivermead Assessment of Somatosensory Performance (RASP): Standardization and reliability data. Clin Rehabil. 2002;16:523–33.

18. Otaka E, Otaka Y, Kasuga S, Nishimoto A, Yamazaki K, Kawakami M, et al. Reliability of the thumb localizing test and its validity against quantitative measures with a robotic device in patients with hemiparetic stroke. PLoS One. 2020;15 7 July:1–13.

19. Bertrand AM, Fournier K, Wick Brasey MG, Kaiser ML, Frischknecht R, Diserens K. Reliability of maximal grip strength measurements and grip strength recovery following a stroke. J Hand Ther. 2015;28:356–63.

20. Fugl-Meyer AR, Jääskö L, Leyman I, Olsson S. The post-stroke hemiplegic patient. 1. a method for evaluation of physical performance. Scandinavian journal of rehabilitation medicine. 1975;7:13.

21. Hsieh YW, Hsueh IP, Chou YT, Sheu CF, Hsieh CL, Kwakkel G. Development and validation of a short form of the Fugl-Meyer motor scale in patients with stroke. Stroke. 2007;38:3052–4.

22. Bohannon RW, Smith MB. Interrater Reliability of a Modified Ashworth Scale of Muscle Spasticity. Phys Ther. 1987;67:206–7.

23. Collin C, Wade D. Assessing motor impairment after stroke: A pilot reliability study. J Neurol Neurosurg Psychiatry. 1990;53:576–9.

24. Demeurisse G, Demol O, Robaye E. Motor evaluation in vascular hemiplegia. Eur Neurol. 1980;19:382–9.

25. Lyle RC. A performance test for assessment of upper limb function in physical rehabilitation treatment and research. Int J Rehabil Res. 1981;4:483–92.

26. Mathiowetz V, Volland G, Kashman N, Weber K. Adult Norms for the Box and Block Test of Manual Dexterity. Am J Occup Ther. 1985;39:386–91.

27. Mathiowetz V, Weber K, Kashman N, Volland G. Adult Norms for the Nine Hole Peg Test of Finger Dexterity. Occup Ther J Res. 1985;5:24–38.

28. Cocchini G, Beschin N, Jehkonen M. The Fluff Test: A simple task to assess body representation neglect. Neuropsychol Rehabil. 2001;11:17–31.

29. Cocchini G, Beschin N. The Fluff test: Improved scoring system to account for different degrees of contralesional and ipsilesional personal neglect in brain damaged patients. Neuropsychol Rehabil. 2020;32:69–83.

30. Nasreddine ZS, Phillips NA, Bédirian V, Charbonneau S, Whitehead V, Collin I, et al. The Montreal Cognitive Assessment, MoCA: A Brief Screening Tool For Mild Cognitive Impairment. J Am Geriatr Soc. 2005;53:695–9.

31. Bickerton WL, Samson D, Williamson J, Humphreys GW. Separating Forms of Neglect Using the Apples Test Validation and Functional Prediction in Chronic and Acute Stroke. Neuropsychology. 2011;25:567–80.

32. Chechlacz M, Rotshtein P, Bickerton WL, Hansen PC, Deb S, Humphreys GW. Separating neural correlates of allocentric and egocentric neglect: Distinct cortical sites and common white matter disconnections. Cogn Neuropsychol. 2010;27:277–303.

33. Zigmond AS, Snaith RP. The Hospital Anxiety and Depression Scale. Acta Psychiatr Scand. 1983;67:361–70.

34. Python G, Bischof S, Probst M, Laganaro M. TICSf-12 : Une épreuve de dépistage des troubles de la compréhension. Aphasie domaines Assoc. 2013;2:70–7.

35. Luzzatti C, Willmes K, Bisiacchi P, De Bleser R, Faglia L, Mazzucchi A, et al. L’Aachener Aphasie Test (AAT): proprietà psicometriche della versione italiana. Arch Psicol Neurol Psichiatr. 1987;48:25–62.

36. Reitan RM. Validity of the Trail Making Test as an Indicator of Organic Brain Damage. Percept Mot Skills. 1958;8:271–6.

37. D’Elia L, Satz P, Uchiyama C, White T. Color Trails Test. Lutz, FL: Psychological Assessment Resources Inc. 1996.

38. Wilson BA, Greenfield E, Clare L, Baddeley A, Cockburn J, Watson P, et al. Rivermead Behavioural Memory Test | Third Edition. 2008.

39. Wechsler D. Wechsler Adult Intelligence Scale--Fourth Edition (WAIS IV). 2008.

40. Wechsler D. Wechsler Adult Intelligence Scale--Third Edition (WAIS-III). 1997.

41. Monaco M, Costa A, Caltagirone C, Carlesimo GA. Forward and backward span for verbal and visuo-spatial data: standardization and normative data from an Italian adult population. Neurol Sci. 2013;34:749–54.

42. Monaco M, Costa A, Caltagirone C, Carlesimo GA. Erratum to: Forward and backward span for verbal and visuo-spatial data: standardization and normative data from an Italian adult population. Neurol Sci. 2015;36:345–7.

43. Mahieux-Laurent F, Fabre C, Galbrun E, Dubrulle A, Moroni C. Validation of a brief screening scale evaluating praxic abilities for use in memory clinics. Evaluation in 419 controls, 127 mild cognitive impairment and 320 demented patients. Rev Neurol (Paris). 2009;165:560–7.
