# Supplementary material for: Evaluation of upper limb perception after stroke with the new Affected Limb Perception Questionnaire (ALPQ): a study protocol
Source: BMC Neurol. 2024 Jun 11;24:196. doi: 10.1186/s12883-024-03648-6 (PMC11165777; doi:10.1186/s12883-024-03648-6)
Supplement: Supplementary file 2 — Supplementary Material 2. [file 12883_2024_3648_MOESM2_ESM.pdf]

ADDITIONAL MATERIAL

Additional file 2

This document provides the b-ALPQ, in French:

- Version: **bALPQ-UL-STK v1.0** (dated 15 February 2022), where UL stands for "upper limb", and STK stands for "stroke".

The full documentation can be found on OSF: https://osf.io/s7e2x.<br>For correct administration of the ALPQ, it is necessary to follow the instructions and a short training by contacting the corresponding authors.

QUESTIONNAIRE SUR LA PERCEPTION DU MEMBRE AFFECTÉ
– MEMBRE SUPÉRIEUR –

binary ALPQ

**Date** d'administration du questionnaire (jj-mm-yyyy) :		**Etiquette patient :**	
**Nom de l'examinateur :**			

**Bras/Main évalué(e) ***	◯ GAUCHE	◯ DROIT(E)

** Membre supérieur affecté par la lésion (par exemple, dans le cas d'une lésion corticale, il s'agira du membre supérieur contralésionnel à la lésion corticale, mais (!) dans le cas d'une lésion au niveau du cervelet, le membre supérieur affecté est alors celui ipsilésionnel à la lésion du cervelet).*

**Le patient peut-il porter son regard sur son membre supérieur affecté (spontanément ou sur stimulation verbale/sonore) ?**<br>*(!) aucune stimulation tactile ne doit être administrée*	◯ OUI, spontanément<br>◯ OUI, seulement sur stimulation verbale et/ou sonore<br>◯ NON

« L'objectif de ce questionnaire est de comprendre la perception que vous avez de votre corps depuis l'AVC. »

*Si le patient reporte une impression positive à une question (i.e. répond 'oui') : demander **systématiquement** s'il a encore ce ressenti « actuellement, au cours de la journée et de la nuit » (i.e. des dernières 24h).*

- *Si 'oui' (encore ce ressenti),*
  - *Indiquer 'oui' sur le questionnaire*
  - *Dans la zone de commentaire rajouter toutes précisions utiles.*

- *Si 'non' (n'a plus ce ressenti, i.e. si le patient indique avoir ressenti quelque chose au début de l'AVC mais plus maintenant) :*
  - *Indiquer 'non' sur le questionnaire (i.e. qu'il ne l'a plus).*
  - *Dans la zone de commentaire, indiquer la réponse exacte du patient / quand il avait l'impression, et autres détails pertinents (e.g. 'il a eu cette impression durant l'AVC et le jour suivant, mais ne l'a plus depuis').*

1. Douleurs - Avez-vous des douleurs au niveau de ce bras/cette main ? ◯ OUI ◯ NON

Précisions et/ou commentaires :

2. Anosognosie de l'hémiplégie - Avez-vous des difficultés à bouger ce bras/cette main ? ◯ OUI ◯ NON

Précisions et/ou commentaires :

Question 3. à poser si réponse 'OUI' à question 2. Sinon cocher 'n/a' et passer directement à la question 4.

3. Anosodiaphorie de l'hémiplégie
Et est-ce que ces difficultés vous affectent émotionnellement? ◯ OUI ◯ NON ◯ n/a

Précisions et/ou commentaires :

4. Hémiasomatognosie
Avez-vous l'impression que ce bras/cette main ne fait pas partie de votre propre corps, qu'il/elle ne vous appartient pas ? ◯ OUI ◯ NON

Précisions et/ou commentaires :

5. Somatoparaphrénie
a) Avez-vous l'impression que ce bras/cette main appartient à quelqu'un d'autre ? ◯ OUI ◯ NON

Si oui, précisez à qui le bras/la main appartient :

Précisions et/ou commentaires :

b) Avez-vous l'impression que ce bras/cette main correspond à quelque chose d'inhumain ? ◯ OUI ◯ NON
Par exemple, un objet.

Si oui, précisez à quoi le bras/la main correspond :

Précisions et/ou commentaires :

6. Personnification du membre atteint - Avez-vous l'impression que ce bras/cette main a une personnalité ou une identité ?
Par exemple, il vous arrive de lui donner un nom, un surnom ou un diminutif.

○ OUI ○ NON

Si oui : précisez :

Précisions et/ou commentaires :

7. Illusion de modification des caractéristiques physiques

a) Avez-vous l'impression que ce bras/cette main a changé de température c'est-à-dire que vous le/la percevez plus chaud(e) ou plus froid(e) ?
Note : il s'agit bien entendu d'un changement depuis l'AVC/depuis cette hospitalisation.

○ OUI ○ NON

Si oui, précisez : ○ + chaud(e) ○ + froid(e)

Précisions et/ou commentaires :

b) Avez-vous l'impression que ce bras/cette main a changé de poids, c'est-à-dire que vous le/la percevez plus lourd(e) ou plus léger(e) ?
Note : il s'agit bien entendu d'un changement depuis l'AVC/depuis cette hospitalisation.

○ OUI ○ NON

Si oui, précisez : ○ + lourd(e) ○ + léger(e)

Précisions et/ou commentaires :

c) Avez-vous l'impression que ce bras/cette main a changé de longueur, c'est-à-dire que vous le/la percevez plus long(ue) ou plus court(e) ?
Note : il s'agit bien entendu d'un changement depuis l'AVC/depuis cette hospitalisation.

○ OUI ○ NON

Si oui, précisez : ○ + long(ue) ○ + court(e)

Précisions et/ou commentaires :

8. Mouvements illusoires - Avez-vous l'impression que ce bras/cette main bouge alors qu'il/elle ne bouge pas en réalité ?
Par exemple : vous avez l'impression que ce bras/cette main bouge, mais lorsque vous les regardez pour vérifier, ils ne bougent pas en réalité.

◯ OUI ◯ NON

Si oui : précisez : *Cocher la/les réponse(s) correspondante(s)*

- ☐ Cette impression est un ressenti sensoriel (vous expérimentez une sensation de mouvement)
- ☐ Cette impression est une représentation mentale (vous visualisez le mouvement dans votre tête)
- ☐ Cette impression de mouvement a lieu quand vous avez l'intention de mettre ce bras/cette main en mouvement
- ☐ Cette impression de mouvement a lieu quand vous n'avez pas l'intention de mettre ce bras/cette main en mouvement)

Précisions et/ou commentaires :

9. Membre surnuméraire/sous-numéraire - Avez-vous l'impression d'avoir *plus de* deux bras et/ou deux mains ou avez-vous l'impression d'avoir *moins de* deux bras et/ou deux mains ?

◯ OUI ◯ NON

Si oui : précisez combien de bras/mains, et où sont-ils/elles situé(e)s (zone du corps et côté du corps) :

Précisions et/ou commentaires :

10. Membre supérieur détaché du reste du corps - Avez-vous l'impression que ce bras/cette main est détaché(e) du reste de votre corps (une impression de corps fractionné) ?

◯ OUI ◯ NON

Si oui : décrivez cette impression :

Précisions et/ou commentaires :

11. Misoplégie - Avez-vous une attitude malveillante envers ce bras/cette main ?
Par exemple : vous ressentez de la colère, du mépris, de l'agressivité (verbale et/ou physique) ou de la haine envers ce bras/cette main.

◯ OUI ◯ NON

Précisions et/ou commentaires :

12. Main étrangère/Mouvements involontaires - Est-ce que ce bras/cette main réalise parfois des mouvements involontaires, non désirés ? Par exemple : cette main bouge alors que vous n'avez pas décidé de la mettre en mouvement.

◯ OUI ◯ NON

Si oui : précisez s'il s'agit : *Cocher la/les réponse(s) correspondante(s)*

- ☐ De mouvements de lévitation (le bras/la main a tendance à s'élever vers le haut).
- ☐ De mouvements de préhension, manipulation ou d'agrippement d'objets (la main a tendance à prendre, manipuler ou s'agripper aux objets).
- ☐ D'un conflit entre les deux mains : une main réalise une activité et l'autre main vient troubler l'activité en cours en réalisant par exemple une activité contraire (par exemple une main boutonne une chemise et l'autre main vient déboutonner cette même chemise l'instant d'après).
- ☐ *De mouvements (ataxiques) qui manquent de précision (au moment de prendre un objet, la main s'oriente à côté de la cible ce qui nécessite d'ajuster le mouvement de préhension par rapport à l'objet).
- ☐ *De mouvements maladroits ou interrompus liés à une faiblesse musculaire (par exemple objet lâché par manque de force).
- ☐ Autre (si autre, précisez ci-dessous).

** ces suggestions ne sont pas considérées comme mouvements involontaires mais doivent tout de même être cochées si présentes.*

Précisions et/ou commentaires :

13. Avez-vous d'autres sensations/impressions au niveau des bras/des mains, dont nous n'avons pas parlé ?

+ Autres sensations/impressions rapportées spontanément par le patient

Précisez pour chaque sensation/impression si elle se réfère au membre affecté ou non-affecté.
