# Supplementary material for: Evaluation of upper limb perception after stroke with the new Affected Limb Perception Questionnaire (ALPQ): a study protocol
Source: BMC Neurol. 2024 Jun 11;24:196. doi: 10.1186/s12883-024-03648-6 (PMC11165777; doi:10.1186/s12883-024-03648-6)
Supplement: Supplementary file 3 — Supplementary Material 3. [file 12883_2024_3648_MOESM3_ESM.pdf]

ADDITIONAL MATERIAL
Additional file 3

This document list all questions of the VAS-ALPQ, in French (page 2 of this .pdf) and in Italian (page 27 of this .pdf):

- Version: **ALPQ-UL-STK v1.0 (FR)** (dated 09 November 2021) and **v1.0 (IT)** (dated 22 March 2022), where UL stands for "upper limb", and STK stands for "stroke".
- The wording "HAUT"/"ALTO" and "BAS"/"BASSO" refer to the sentence written at the top ("haut"/"alto") and bottom ("bas"/basso") of each VAS scale.

The full documentation, including the scales of the VAS-ALPQ as well as the instruction manual, can be found on OSF: https://osf.io/s7e2x.<br><br>For correct administration of the ALPQ, it is necessary to follow the instruction manual and a short training by contacting the corresponding authors.

ALPQ

Affected Limb Perception Questionnaire

QUESTIONNAIRE SUR LA PERCEPTION DU MEMBRE AFFECTÉ

ALPQ-UL-STK
Version 1.0 (FR)
(09 November 2021)

CONTRÔLE	**0.a.**<br>**FATIGUE**

Haut	*Fatigue maximale*
Bas	*Pas de fatigue*

CONTRÔLE	**0.b.**<br><br>**TRISTESSE**

Haut	*Tristesse maximale*
Bas	*Pas de tristesse*

CONTRÔLE	**0.c.**<br>**ANXIÉTÉ**

Haut	*Anxiété maximale*
Bas	*Pas d'anxiété*

MEMBRE LE PLUS AFFECTÉ	**1**<br>**DOULEURS**

Haut (14)	*J'ai des douleurs au niveau de ce bras/cette main.*
Bas (0)	*Je n'ai pas de douleurs au niveau de ce bras/cette main.*

Si douleurs (si score > 0)

Intensité (phase aiguë): *Quelle est l'intensité moyenne de ces douleurs? (cocher)*
Intensité (phase sub-aiguë): *Ces 7 derniers jours, quelle a été l'intensité moyenne de ces douleurs ? (cocher)*

○ Douleurs légères
○ Douleurs modérées
○ Douleurs sévères

Fréquence (phase aiguë): *A quelle fréquence ces douleurs sont-elles présentes? (cocher)*
Fréquence (phase sub-aiguë/chronique): *A quelle fréquence ces douleurs ont-elles été présentes ces 7 derniers jours ? (cocher)*

○ Rarement : moins d'une fois par jour
○ Souvent : une à plusieurs fois par jour
○ Continuellement : tout le temps

Si Rarement ou Souvent:
Ces douleurs sont-elles plus marquées à un moment particulier de la journée (par exemple le matin au réveil, en fin de matinée, en fin de journée ou pendant la nuit) ?

Commentaire de l'expérimentateur:
E.g. noter si les réponses du patient semblent inventées et si oui sur quelle(s) question(s)

Autres sensations rapportées spontanément par le patient
Préciser pour chaque sensation/impression si elle se réfère au membre ipsilésionnel ou contralésionnel.

MEMBRE LE PLUS AFFECTÉ	**2**<br>**ANOSOGNOSIE DE L'HÉMIPLÉGIE**

Haut (0)	*J'ai des difficultés à bouger ce bras/cette main.*
Bas (14)	*Je n'ai pas de difficultés à bouger ce bras/cette main.*

Si difficultés (si score <14)

Intensité (phase aiguë): *Quelle est l'intensité moyenne de ces difficultés? (cocher)*
Intensité (phase sub-aiguë): *Ces 7 derniers jours, quelle a été l'intensité moyenne de ces difficultés? (cocher)*

- ○ Difficultés légères
- ○ Difficultés modérées
- ○ Difficultés sévères

Fréquence (phase aiguë): *A quelle fréquence ces difficultés sont-elles présentes? (cocher)*
Fréquence (phase sub-aiguë/chronique): *A quelle fréquence ces difficultés ont-elles été présentes ces 7 derniers jours ? (cocher)*

- ○ Rarement : moins d'une fois par jour
- ○ Souvent : une à plusieurs fois par jour
- ○ Continuellement : tout le temps

Si Rarement ou Souvent:
Ces difficultés sont-elles plus marquées à un moment particulier de la journée (par exemple le matin au réveil, en fin de matinée, en fin de journée ou pendant la nuit) ?

Commentaire de l'expérimentateur:
E.g. noter si les réponses du patient semblent inventées et si oui sur quelle(s) question(s)

Autres sensations rapportées spontanément par le patient
Préciser pour chaque sensation/impression si elle se réfère au membre ipsilésionnel ou contralésionnel.

MEMBRE LE PLUS AFFECTÉ	**3**<br>**ANOSODIAPHORIE DE L'HÉMIPLÉGIE**

VAS CONTRA.3 à poser uniquement si VAS CONTRA.2 <14. Sinon passer directement au VAS suivant.

Haut (0)	*Ces difficultés m'affectent émotionnellement.*<br>Répéter qu'il s'agit des difficultés motrices évoquées précédemment
Bas (14)	*Ces difficultés ne m'affectent pas émotionnellement.*<br>Répéter qu'il s'agit des difficultés motrices évoquées précédemment

Si affecté (si score <14)

Intensité (phase aiguë): *A quelle intensité, en moyenne, êtes-vous affecté ·e émotionnellement par ces difficultés ? (cocher)*
Intensité (phase sub-aiguë): *Ces 7 derniers jours, à quelle intensité, en moyenne, avez-vous été affecté·e émotionnellement par ces difficultés ? (cocher)*

○ Affecté·e légèrement
○ Affecté·e modérément
○ Affecté·e sévèrement

Fréquence (phase aiguë): *A quelle fréquence vous sentez-vous affecté ·e émotionnellement par ces difficultés? (cocher)*
Fréquence (phase sub-aiguë/chronique): *A quelle fréquence vous êtes-vous senti affecté·e émotionnellement par ces difficultés ces 7 derniers jours ? (cocher)*

○ Rarement : moins d'une fois par jour
○ Souvent : une à plusieurs fois par jour
○ Continuellement : tout le temps

Si Rarement ou Souvent:
Est-ce que ce ressenti émotionnel est plus marqué à un moment particulier de la journée (par exemple le matin au réveil, en fin de matinée, en fin de journée ou pendant la nuit)?

Commentaire de l'expérimentateur:
E.g. noter si les réponses du patient semblent inventées et si oui sur quelle(s) question(s)

Autres sensations rapportées spontanément par le patient
Préciser pour chaque sensation/impression si elle se réfère au membre ipsilésionnel ou contralésionnel.

MEMBRE LE PLUS AFFECTÉ	**4**<br>**HÉMIASOMATOGNOSIE**

Haut (14)	J'ai l'impression que ce bras/cette main ne fait pas partie de mon propre corps, qu'il/elle ne m'appartient pas.
Bas (0)	J'ai l'impression que ce bras/cette main fait partie de mon propre corps, qu'il/elle m'appartient.

Si score > 0

Intensité (phase aiguë): *Quelle est l'intensité moyenne de cette impression? (cocher)*
Intensité (phase sub-aiguë): *Ces 7 derniers jours, quelle a été l'intensité moyenne de cette impression ? (cocher)*

- ○ Impression légère
- ○ Impression modérée
- ○ Impression forte

Fréquence (phase aiguë): *A quelle fréquence cette impression est-elle présente? (cocher)*
Fréquence (phase sub-aiguë/chronique): *A quelle fréquence cette impression a-t-elle été présente ces 7 derniers jours ? (cocher)*

- ○ Rarement : moins d'une fois par jour
- ○ Souvent : une à plusieurs fois par jour
- ○ Continuellement : tout le temps

Si Rarement ou Souvent:
Cette impression est-elle plus marquée à un moment particulier de la journée (par exemple le matin au réveil, en fin de matinée, en fin de journée ou pendant la nuit) ?

Commentaire de l'expérimentateur:
E.g. noter si les réponses du patient semblent inventées et si oui sur quelle(s) question(s)

Autres sensations rapportées spontanément par le patient
Préciser pour chaque sensation/impression si elle se réfère au membre ipsilésionnel ou contralésionnel.

MEMBRE LE PLUS AFFECTÉ	**5.a.** **SOMATOPARAPHRÉNIE (1/2)**
Haut (14)	J'ai l'impression que ce bras/cette main appartient à quelqu'un d'autre.
Bas (0)	Je n'ai pas l'impression que ce bras/cette main appartient à quelqu'un d'autre

Si score > 0

Si impression que le bras/la main appartient à quelqu'un d'autre, précisez: "à qui ce bras/cette main appartient?"

Intensité (phase aiguë): *Quelle est l'intensité moyenne de cette impression? (cocher)*
Intensité (phase sub-aiguë): *Ces 7 derniers jours, quelle a été l'intensité moyenne de cette impression ? (cocher)*

- ○ Impression légère
- ○ Impression modérée
- ○ Impression forte

Fréquence (phase aiguë): *A quelle fréquence cette impression est-elle présente? (cocher)*
Fréquence (phase sub-aiguë/chronique): *A quelle fréquence cette impression a-t-elle été présente ces 7 derniers jours ? (cocher)*

- ○ Rarement : moins d'une fois par jour
- ○ Souvent : une à plusieurs fois par jour
- ○ Continuellement : tout le temps

Si Rarement ou Souvent:
Cette impression est-elle plus marquée à un moment particulier de la journée (par exemple le matin au réveil, en fin de matinée, en fin de journée ou pendant la nuit) ?

Commentaire de l'expérimentateur:
E.g. noter si les réponses du patient semblent inventées et si oui sur quelle(s) question(s)

Autres sensations rapportées spontanément par le patient
Préciser pour chaque sensation/impression si elle se réfère au membre ipsilésionnel ou contralésionnel.

MEMBRE LE PLUS AFFECTÉ	**5.b.**<br>**SOMATOPARAPHRÉNIE (2/2)**

Haut (14)	J'ai l'impression que ce bras/cette main correspond à quelque chose d'inhumain<br>*Par exemple, un objet.*
Bas (0)	Je n'ai pas l'impression que ce bras/cette main correspond à quelque chose d'inhumain

Si score > 0

Si impression que le bras/la main correspond à quelque chose d'inhumain, précisez: "à quoi ce bras/cette main correspond?"

Intensité (phase aiguë): *Quelle est l'intensité moyenne de cette impression? (cocher)*
Intensité (phase sub-aiguë): *Ces 7 derniers jours, quelle a été l'intensité moyenne de cette impression ? (cocher)*

- ○ Impression légère
- ○ Impression modérée
- ○ Impression forte

Fréquence (phase aiguë): *A quelle fréquence cette impression est-elle présente? (cocher)*
Fréquence (phase sub-aiguë/chronique): *A quelle fréquence cette impression a-t-elle été présente ces 7 derniers jours ? (cocher)*

- ○ Rarement : moins d'une fois par jour
- ○ Souvent : une à plusieurs fois par jour
- ○ Continuellement : tout le temps

Si Rarement ou Souvent:
Cette impression est-elle plus marquée à un moment particulier de la journée (par exemple le matin au réveil, en fin de matinée, en fin de journée ou pendant la nuit) ?

Commentaire de l'expérimentateur:
E.g. noter si les réponses du patient semblent inventées et si oui sur quelle(s) question(s)

Autres sensations rapportées spontanément par le patient
Préciser pour chaque sensation/impression si elle se réfère au membre ipsilésionnel ou contralésionnel.

MEMBRE LE PLUS AFFECTÉ	**6**<br>**PERSONNIFICATION DU MEMBRE ATTEINT**

Haut (14)	J'ai l'impression que ce bras/cette main a une personnalité ou une identité<br>*Par exemple: il m'arrive de lui donner un nom, un surnom ou un diminutif.*
Bas (0)	Je n'ai pas l'impression que ce bras/cette main a une personnalité ou une identité

Si score > 0

Si impression que ce bras/cette main a une personnalité ou une identité, précisez quelle personnalité/identité :

Intensité (phase aiguë): *Quelle est l'intensité moyenne de cette impression? (cocher)*
Intensité (phase sub-aiguë): *Ces 7 derniers jours, quelle a été l'intensité moyenne de cette impression ? (cocher)*

- ○ Impression légère
- ○ Impression modérée
- ○ Impression forte

Fréquence (phase aiguë): *A quelle fréquence cette impression est-elle présente? (cocher)*
Fréquence (phase sub-aiguë/chronique): *A quelle fréquence cette impression a-t-elle été présente ces 7 derniers jours ? (cocher)*

- ○ Rarement : moins d'une fois par jour
- ○ Souvent : une à plusieurs fois par jour
- ○ Continuellement : tout le temps

Si Rarement ou Souvent:
Cette impression est-elle plus marquée à un moment particulier de la journée (par exemple le matin au réveil, en fin de matinée, en fin de journée ou pendant la nuit) ?

Commentaire de l'expérimentateur:
E.g. noter si les réponses du patient semblent inventées et si oui sur quelle(s) question(s)

Autres sensations rapportées spontanément par le patient
Préciser pour chaque sensation/impression si elle se réfère au membre ipsilésionnel ou contralésionnel.

MEMBRE LE PLUS AFFECTÉ	**7.a.**<br><br>**ILLUSION DE MODIFICATION DES CARACTÉRISTIQUES PHYSIQUES (TEMPÉRATURE)**

Haut (14)	J'ai l'impression que ce bras/cette main a changé de température<br>*C'est-à-dire que vous le/la percevez plus chaud(e) ou plus froid(e)*<br>*Note (phase aiguë): il s'agit bien entendu d'un changement depuis l'AVC/depuis cette hospitalisation.*<br>*Note (phase sub-aiguë): il s'agit bien entendu d'un changement depuis l'AVC/depuis cette hospitalisation, et de votre ressenti en moyenne au cours des 7 derniers jours.*
Bas (0)	J'ai l'impression que ce bras/cette main n'a pas changé de température

Si score > 0

Si impression que ce bras/cette main a changé de température, précisez :

○ Plus chaud(e)
○ Plus froid(e)

Quelle partie du corps est concernée? (cocher la/les réponse(s) correspondante(s)) :

☐ Bras ☐ Avant-Bras ☐ Main ☐ Doigts

Précisez si besoin:

Intensité (phase aiguë): *Quelle est l'intensité moyenne de cette impression? (cocher)*
Intensité (phase sub-aiguë): *Ces 7 derniers jours, quelle a été l'intensité moyenne de cette impression ? (cocher)*

○ Impression légère
○ Impression modérée
○ Impression forte

Fréquence (phase aiguë): *A quelle fréquence cette impression est-elle présente? (cocher)*
Fréquence (phase sub-aiguë/chronique): *A quelle fréquence cette impression a-t-elle été présente ces 7 derniers jours ? (cocher)*

○ Rarement : moins d'une fois par jour
○ Souvent : une à plusieurs fois par jour
○ Continuellement : tout le temps

Si Rarement ou Souvent:
Cette impression est-elle plus marquée à un moment particulier de la journée (par exemple le matin au réveil, en fin de matinée, en fin de journée ou pendant la nuit) ?

Commentaire de l'expérimentateur:
E.g. noter si les réponses du patient semblent inventées et si oui sur quelle(s) question(s)

Autres sensations rapportées spontanément par le patient
Préciser pour chaque sensation/impression si elle se réfère au membre ipsilésionnel ou contralésionnel.

MEMBRE LE PLUS AFFECTÉ	**7.b.**<br>**ILLUSION DE MODIFICATION DES CARACTÉRISTIQUES PHYSIQUES (POIDS)**

Haut (14)	J'ai l'impression que ce bras/cette main a changé de poids<br>*C'est-à-dire que vous le/la percevez plus lourd(e) ou plus léger(e)*<br>*Note (phase aiguë): il s'agit bien entendu d'un changement depuis l'AVC/depuis cette hospitalisation.*<br>*Note (phase sub-aiguë): il s'agit bien entendu d'un changement depuis l'AVC/depuis cette hospitalisation, et de votre ressenti en moyenne au cours des 7 derniers jours.*
Bas (0)	J'ai l'impression que ce bras/cette main n'a pas changé de poids

Si score > 0

Si impression que ce bras/cette main a changé de poids, précisez :

○ Plus lourd(e)
○ Plus léger(e)

Quelle partie du corps est concernée? (cocher la/les réponse(s) correspondante(s)) :

☐ Bras ☐ Avant-Bras ☐ Main ☐ Doigts

Précisez si besoin:

Intensité (phase aiguë): *Quelle est l'intensité moyenne de cette impression? (cocher)*
Intensité (phase sub-aiguë): *Ces 7 derniers jours, quelle a été l'intensité moyenne de cette impression ? (cocher)*

○ Impression légère
○ Impression modérée
○ Impression forte

Fréquence (phase aiguë): *A quelle fréquence cette impression est-elle présente? (cocher)*
Fréquence (phase sub-aiguë/chronique): *A quelle fréquence cette impression a-t-elle été présente ces 7 derniers jours ? (cocher)*

○ Rarement : moins d'une fois par jour
○ Souvent : une à plusieurs fois par jour
○ Continuellement : tout le temps

Si Rarement ou Souvent:
Cette impression est-elle plus marquée à un moment particulier de la journée (par exemple le matin au réveil, en fin de matinée, en fin de journée ou pendant la nuit) ?

Commentaire de l'expérimentateur:
E.g. noter si les réponses du patient semblent inventées et si oui sur quelle(s) question(s)

Autres sensations rapportées spontanément par le patient
Préciser pour chaque sensation/impression si elle se réfère au membre ipsilésionnel ou contralésionnel.

MEMBRE LE PLUS AFFECTÉ	**7.c.**<br><br>**ILLUSION DE MODIFICATION DES CARACTÉRISTIQUES PHYSIQUES (LONGUEUR)**

Haut (14)	J'ai l'impression que ce bras/cette main a changé de longueur<br>*C'est-à-dire que vous le/la percevez plus long(ue) ou plus court(e)*<br>*Note (phase aiguë): il s'agit bien entendu d'un changement depuis l'AVC/depuis cette hospitalisation.*<br>*Note (phase sub-aiguë): il s'agit bien entendu d'un changement depuis l'AVC/depuis cette hospitalisation, et de votre ressenti en moyenne au cours des 7 derniers jours.*
Bas (0)	J'ai l'impression que ce bras/cette main n'a pas changé de longueur

Si score > 0

Si impression que ce bras/cette main a changé de longueur, précisez :

○ Plus long(ue)
○ Plus court(e)

Quelle partie du corps est concernée? (cocher la/les réponse(s) correspondante(s)) :

☐ Bras ☐ Avant-Bras ☐ Main ☐ Doigts

Précisez si besoin:

Intensité (phase aiguë): *Quelle est l'intensité moyenne de cette impression? (cocher)*
Intensité (phase sub-aiguë): *Ces 7 derniers jours, quelle a été l'intensité moyenne de cette impression ? (cocher)*

○ Impression légère
○ Impression modérée
○ Impression forte

Fréquence (phase aiguë): *A quelle fréquence cette impression est-elle présente? (cocher)*
Fréquence (phase sub-aiguë/chronique): *A quelle fréquence cette impression a-t-elle été présente ces 7 derniers jours ? (cocher)*

○ Rarement : moins d'une fois par jour
○ Souvent : une à plusieurs fois par jour
○ Continuellement : tout le temps

Si Rarement ou Souvent:
Cette impression est-elle plus marquée à un moment particulier de la journée (par exemple le matin au réveil, en fin de matinée, en fin de journée ou pendant la nuit) ?

Commentaire de l'expérimentateur:
E.g. noter si les réponses du patient semblent inventées et si oui sur quelle(s) question(s)

Autres sensations rapportées spontanément par le patient
Préciser pour chaque sensation/impression si elle se réfère au membre ipsilésionnel ou contralésionnel.

MEMBRE LE PLUS AFFECTÉ	**8** **MOUVEMENTS ILLUSOIRES**

Haut (14)	*J'ai l'impression que ce bras/cette main bouge alors qu'il/elle ne bouge pas en réalité.* *Par exemple : j'ai l'impression que ce bras/cette main bouge, mais lorsque je les regarde pour vérifier, ils ne bougent pas en réalité.*
Bas (0)	*J'ai l'impression que ce bras/cette main bouge uniquement lorsqu'il/elle bouge en réalité.*

Si score > 0

Si impression de mouvements illusoires, précisez (cocher la/les réponse(s) correspondante(s)) :

☐ Cette impression est un ressenti sensoriel (vous expérimentez une sensation de mouvement)
☐ Cette impression est une représentation mentale (vous visualisez le mouvement dans votre tête)
☐ Cette impression de mouvement a lieu quand vous avez l'intention de mettre ce bras/cette main en mouvement
☐ Cette impression de mouvement a lieu quand vous n'avez pas l'intention de mettre ce bras/cette main en mouvement)

Précisions et/ou commentaires :

Intensité (phase aiguë): *Quelle est l'intensité moyenne de cette impression? (cocher)*
Intensité (phase sub-aiguë): *Ces 7 derniers jours, quelle a été l'intensité moyenne de cette impression ? (cocher)*

○ Impression légère
○ Impression modérée
○ Impression forte

Fréquence (phase aiguë): *A quelle fréquence cette impression est-elle présente? (cocher)*
Fréquence (phase sub-aiguë/chronique): *A quelle fréquence cette impression a-t-elle été présente ces 7 derniers jours ? (cocher)*

○ Rarement : moins d'une fois par jour
○ Souvent : une à plusieurs fois par jour
○ Continuellement : tout le temps

Si Rarement ou Souvent:
Cette impression est-elle plus marquée à un moment particulier de la journée (par exemple le matin au réveil, en fin de matinée, en fin de journée ou pendant la nuit) ?

Commentaire de l'expérimentateur:
E.g. noter si les réponses du patient semblent inventées et si oui sur quelle(s) question(s)

Autres sensations rapportées spontanément par le patient
Préciser pour chaque sensation/impression si elle se réfère au membre ipsilésionnel ou contralésionnel.

MEMBRE LE PLUS AFFECTÉ	**9**<br>**MEMBRE SURNUMÉRAIRE/SOUS-NUMÉRAIRE**

Haut (14)	J'ai l'impression d'avoir plus de deux bras et/ou deux mains ou j'ai l'impression d'avoir moins de deux bras et/ou deux mains.
Bas (0)	J'ai l'impression que mon corps est constitué de deux bras et deux mains.

Si score > 0

Si impression d'avoir plus ou moins de deux bras et/ou deux mains, précisez combien de bras/mains, et où sont-ils/elles situés (zone du corps et côté du corps) ?

Intensité (phase aiguë): *Quelle est l'intensité moyenne de cette impression? (cocher)*
Intensité (phase sub-aiguë): *Ces 7 derniers jours, quelle a été l'intensité moyenne de cette impression ? (cocher)*

- ○ Impression légère
- ○ Impression modérée
- ○ Impression forte

Fréquence (phase aiguë): *A quelle fréquence cette impression est-elle présente? (cocher)*
Fréquence (phase sub-aiguë/chronique): *A quelle fréquence cette impression a-t-elle été présente ces 7 derniers jours ? (cocher)*

- ○ Rarement : moins d'une fois par jour
- ○ Souvent : une à plusieurs fois par jour
- ○ Continuellement : tout le temps

Si Rarement ou Souvent:
Cette impression est-elle plus marquée à un moment particulier de la journée (par exemple le matin au réveil, en fin de matinée, en fin de journée ou pendant la nuit) ?

Commentaire de l'expérimentateur:
E.g. noter si les réponses du patient semblent inventées et si oui sur quelle(s) question(s)

Autres sensations rapportées spontanément par le patient
Préciser pour chaque sensation/impression si elle se réfère au membre ipsilésionnel ou contralésionnel.

MEMBRE LE PLUS AFFECTÉ	**10**<br>**MEMBRE SUPÉRIEUR DÉTACHÉ DU RESTE DU CORPS**

Haut (14)	J'ai l'impression que ce bras/cette main est détaché(e) du reste de mon corps<br>*(une impression de corps fractionné)*
Bas (0)	Je n'ai pas l'impression que ce bras/cette main est détaché(e) du reste de mon corps

Si score > 0 *Si impression que le corps est fractionné, décrivez cette impression :*

Intensité (phase aiguë): *Quelle est l'intensité moyenne de cette impression? (cocher)*
Intensité (phase sub-aiguë): *Ces 7 derniers jours, quelle a été l'intensité moyenne de cette impression ? (cocher)*

- ○ Impression légère
- ○ Impression modérée
- ○ Impression forte

Fréquence (phase aiguë): *A quelle fréquence cette impression est-elle présente? (cocher)*
Fréquence (phase sub-aiguë/chronique): *A quelle fréquence cette impression a-t-elle été présente ces 7 derniers jours ? (cocher)*

- ○ Rarement : moins d'une fois par jour
- ○ Souvent : une à plusieurs fois par jour
- ○ Continuellement : tout le temps

Si Rarement ou Souvent:
Cette impression est-elle plus marquée à un moment particulier de la journée (par exemple le matin au réveil, en fin de matinée, en fin de journée ou pendant la nuit) ?

Commentaire de l'expérimentateur:
E.g. noter si les réponses du patient semblent inventées et si oui sur quelle(s) question(s)

Autres sensations rapportées spontanément par le patient
Préciser pour chaque sensation/impression si elle se réfère au membre ipsilésionnel ou contralésionnel.

MEMBRE LE PLUS AFFECTÉ	**11**<br>**MISOPLÉGIE**
Haut (14)	J'ai une attitude malveillante envers ce bras/cette main.<br>*Par exemple : je ressens de la colère, du mépris, de l'agressivité (verbale et/ou physique) ou de la haine envers ce bras/cette main.*
Bas (0)	Je n'ai pas une attitude malveillante envers ce bras/cette main.

Si score > 0

Si présence d'une attitude malveillante, comment s'exprime-t-elle ?

a) A travers un ressenti

Par exemple : je ressens de la colère, du mépris ou de la haine envers ce bras/cette main.

b) A travers un comportement

Par exemple : j'ai tendance à critiquer négativement voire insulter ce bras/cette main, à le repousser, à le taper, à le griffer, à le mordre.

Intensité (phase aiguë): *A quelle intensité, en moyenne, cette attitude malveillante s'exprime-t-elle ? (cocher)*

Intensité (phase sub-aiguë): *Ces 7 derniers jours, à quelle intensité, en moyenne, cette attitude malveillante s'est-elle exprimée ? (cocher)*

○ Légèrement
○ Modérément
○ Fortement

Fréquence (phase aiguë): *A quelle fréquence cette attitude malveillante s'exprime-t-elle? (cocher)*

Fréquence (phase sub-aiguë/chronique): *A quelle fréquence cette attitude malveillante c'est elle exprimée ces 7 derniers jours ? (cocher)*

○ Rarement : moins d'une fois par jour
○ Souvent : une à plusieurs fois par jour
○ Continuellement : tout le temps

Si Rarement ou Souvent:

Cette attitude est-elle plus marquée à un moment particulier de la journée (par exemple le matin au réveil, en fin de matinée, en fin de journée ou pendant la nuit) ?

Commentaire de l'expérimentateur:

E.g. noter si les réponses du patient semblent inventées et si oui sur quelle(s) question(s)

Autres sensations rapportées spontanément par le patient

Préciser pour chaque sensation/impression si elle se réfère au membre ipsilésionnel ou contralésionnel.

MEMBRE LE PLUS AFFECTÉ	**12** **MOUVEMENTS INVOLONTAIRES**

Haut (14)	*Ce bras/cette main réalise parfois des mouvements involontaires, non désirés.* *Par exemple : cette main bouge alors que vous n'avez pas décidé de la mettre en mouvement.*
Bas (0)	*Les mouvements de ce bras/cette main sont volontaires.* *Par exemple : cette main bouge car vous avez décidé de la mettre en mouvement.*

Si score > 0

Si ce bras/cette main a un comportement moteur involontaire, précisez si présence (cocher la/les réponse(s) correspondante(s)) :

☐ De mouvements de lévitation (le bras/la main a tendance à s'élever vers le haut).
☐ De mouvements de préhension, manipulation ou d'agrippement d'objets (la main a tendance à prendre, manipuler ou s'agripper aux objets).

☐ D'un conflit entre les deux mains : une main réalise une activité et l'autre main vient troubler l'activité en cours en réalisant par exemple une activité contraire (par exemple une main boutonne une chemise et l'autre main vient déboutonner cette même chemise l'instant d'après).
☐ * De mouvements (ataxiques) qui manquent de précision (au moment de prendre un objet, la main s'oriente à côté de la cible ce qui nécessite d'ajuster le mouvement de préhension par rapport à l'objet).
☐ * De mouvements maladroits ou interrompus liés à une faiblesse musculaire (par exemple objet lâché par manque de force).
☐ Autre (si autre, précisez ci-dessous).

** ces suggestions ne sont pas considérées comme mouvements involontaires mais doivent tout de même être cochées si présentes.*

Précisions et/ou commentaires :

Intensité (phase aiguë): *Quelle est l'intensité moyenne de ces mouvements involontaires? (cocher)*
Intensité (phase sub-aiguë): *Ces 7 derniers jours, quelle a été l'intensité moyenne de ces mouvements involontaires? (cocher)*

○ Mouvements involontaires légers
○ Mouvements involontaires modérés
○ Mouvements involontaires forts

Fréquence (phase aiguë): *A quelle fréquence ces mouvements involontaires sont-ils présents? (cocher)*
Fréquence (phase sub-aiguë/chronique): *A quelle fréquence ces mouvements involontaires ont-ils été présents ces 7 derniers jours ? (cocher)*

○ Rarement : moins d'une fois par jour
○ Souvent : une à plusieurs fois par jour
○ Continuellement : tout le temps

Si Rarement ou Souvent:
Ces mouvements involontaires apparaissent-ils à un moment particulier de la journée (par exemple le matin au réveil, en fin de matinée, en fin de journée ou pendant la nuit) ?

Commentaire de l'expérimentateur:
E.g. noter si les réponses du patient semblent inventées et si oui sur quelle(s) question(s)

Autres sensations rapportées spontanément par le patient
Préciser pour chaque sensation/impression si elle se réfère au membre ipsilésionnel ou contralésionnel.

MEMBRE LE MOINS AFFECTÉ	**1**<br>**ANOSOGNOSIE DE L'HÉMIPLÉGIE**

Haut (0)	*J'ai des difficultés à bouger ce bras/cette main.*
Bas (14)	*Je n'ai pas de difficultés à bouger ce bras/cette main.*

Si difficultés (si score <14)

Intensité (phase aiguë): *Quelle est l'intensité moyenne de ces difficultés? (cocher)*
Intensité (phase sub-aiguë): *Ces 7 derniers jours, quelle a été l'intensité moyenne de ces difficultés? (cocher)*

- ○ Difficultés légères
- ○ Difficultés modérées
- ○ Difficultés sévères

Fréquence (phase aiguë): *A quelle fréquence ces difficultés sont-elles présentes? (cocher)*
Fréquence (phase sub-aiguë/chronique): *A quelle fréquence ces difficultés ont-elles été présentes ces 7 derniers jours ? (cocher)*

- ○ Rarement : moins d'une fois par jour
- ○ Souvent : une à plusieurs fois par jour
- ○ Continuellement : tout le temps

Si Rarement ou Souvent:
Ces difficultés sont-elles plus marquées à un moment particulier de la journée (par exemple le matin au réveil, en fin de matinée, en fin de journée ou pendant la nuit) ?

Commentaire de l'expérimentateur:
E.g. noter si les réponses du patient semblent inventées et si oui sur quelle(s) question(s)

Autres sensations rapportées spontanément par le patient
Préciser pour chaque sensation/impression si elle se réfère au membre ipsilésionnel ou contralésionnel.

MEMBRE LE MOINS AFFECTÉ	**2**<br>**HÉMIASOMATOGNOSIE**

Haut (14)	J'ai l'impression que ce bras/cette main ne fait pas partie de mon propre corps, qu'il/elle ne m'appartient pas.
Bas (0)	J'ai l'impression que ce bras/cette main fait partie de mon propre corps, qu'il/elle m'appartient.

Si score > 0

Intensité (phase aiguë): *Quelle est l'intensité moyenne de cette impression? (cocher)*
Intensité (phase sub-aiguë): *Ces 7 derniers jours, quelle a été l'intensité moyenne de cette impression ? (cocher)*

- ○ Impression légère
- ○ Impression modérée
- ○ Impression forte

Fréquence (phase aiguë): *A quelle fréquence cette impression est-elle présente? (cocher)*
Fréquence (phase sub-aiguë/chronique): *A quelle fréquence cette impression a-t-elle été présente ces 7 derniers jours ? (cocher)*

- ○ Rarement : moins d'une fois par jour
- ○ Souvent : une à plusieurs fois par jour
- ○ Continuellement : tout le temps

Si Rarement ou Souvent:
Cette impression est-elle plus marquée à un moment particulier de la journée (par exemple le matin au réveil, en fin de matinée, en fin de journée ou pendant la nuit) ?

Commentaire de l'expérimentateur:
E.g. noter si les réponses du patient semblent inventées et si oui sur quelle(s) question(s)

Autres sensations rapportées spontanément par le patient
Préciser pour chaque sensation/impression si elle se réfère au membre ipsilésionnel ou contralésionnel.

MEMBRE LE MOINS AFFECTÉ	**3**<br><br>**SOMATOPARAPHRÉNIE**

Haut (14)	J'ai l'impression que ce bras/cette main appartient à quelqu'un d'autre.
Bas (0)	Je n'ai pas l'impression que ce bras/cette main appartient à quelqu'un d'autre

Si score > 0

Si impression que le bras/la main appartient à quelqu'un d'autre, précisez: "à qui ce bras/cette main appartient?"

Intensité (phase aiguë): *Quelle est l'intensité moyenne de cette impression? (cocher)*
Intensité (phase sub-aiguë): *Ces 7 derniers jours, quelle a été l'intensité moyenne de cette impression ? (cocher)*

- O Impression légère
- O Impression modérée
- O Impression forte

Fréquence (phase aiguë): *A quelle fréquence cette impression est-elle présente? (cocher)*
Fréquence (phase sub-aiguë/chronique): *A quelle fréquence cette impression a-t-elle été présente ces 7 derniers jours ? (cocher)*

- O Rarement : moins d'une fois par jour
- O Souvent : une à plusieurs fois par jour
- O Continuellement : tout le temps

Si Rarement ou Souvent:
Cette impression est-elle plus marquée à un moment particulier de la journée (par exemple le matin au réveil, en fin de matinée, en fin de journée ou pendant la nuit) ?

Commentaire de l'expérimentateur:
E.g. noter si les réponses du patient semblent inventées et si oui sur quelle(s) question(s)

Autres sensations rapportées spontanément par le patient
Préciser pour chaque sensation/impression si elle se réfère au membre ipsilésionnel ou contralésionnel.

AUTRES SENSATIONS

Avez-vous d'autres sensations au niveau des bras/des mains, dont nous n'avons pas parlé ?
Précisez pour chaque sensation/impression si elle se réfère au membre ipsilésionnel ou contralésionnel.

Commentaire de l'expérimentateur:
E.g. noter si certaines sensations reportées par le patient semblent inventées, et si oui quelle(s) sensation(s)

ALPQ

Affected Limb Perception Questionnaire

QUESTIONARIO SULLA PERCEZIONE DELL' ARTO COLPITO

ALPQ-UL-STK
Version 1.0 (IT)
(22 March 2022)

CONTROLLO	**0.A.**<br>**FATICA**

Alto	*Fatica massima*
Basso	*Nessuna fatica*

CONTROLLO	**0.B.**<br>**TRISTEZZA**

Alto	*Tristezza massima*
Basso	*Nessuna tristezza*

CONTROLLO	**0.C.**<br>**ANSIA**

Alto	*Ansia massima*
Basso	*Nessuna ansia*

ARTO PIÙ COLPITO	1<br>DOLORE
Alto (14)	*Sento dei dolori su questo braccio/questa mano*
Basso (0)	*Non sento dolore su questo braccio/questa mano*

Se prova dolore (punteggio > 0)

Intensità (fase acuta): *Quale è l'intensità media di questi dolori? (selezionare)*
Intensità (fase sub-acuta): *Negli ultimi 7 giorni, quale è stata l'intensità media di questi dolori? (selezionare)*

○ Dolori leggeri
○ Dolori moderati
○ Dolori forti

Frequenza (fase acuta): *Con quale frequenza si presentano questi dolori? (selezionare)*
Frequenza (fase sub-acuta/cronica): *Con quale frequenza si sono presentati questi dolori negli ultimi 7 giorni? (selezionare)*

○ Raramente: meno di una volta al giorno
○ Spesso : da una a più volte al giorno
○ Continuamente : tutto il tempo

Se Raramente o Spesso:
Questi dolori sono più marcati in un momento specifico della giornata (per esempio la mattina al risveglio, a fine mattinata, a fine giornata o durante la notte)?

Commenti dello sperimentatore:

Es. Annotare se le risposte del paziente sembrano inventate e se si indicare per quale/i domanda/e

Altre sensazioni riportate spontaneamente dal paziente

Specificare per ogni sensazione/impressione se si riferisce all'arto ipsilesionale o controlesionale.

ARTO PIÙ COLPITO	2<br>ANOSOGNOSIA DELL'EMIPLEGIA

Alto (0)	*Ho difficoltà a muovere questo braccio/questa mano*
Basso (14)	*Non ho difficoltà a muovere questo braccio/questa mano*

Se è presente una difficoltà (punteggio < 14)

Intensità (fase acuta): *Quale è l'intensità media di queste difficoltà? (selezionare)*
Intensità (fase sub-acuta): *Negli ultimi 7 giorni, quale è stata l'intensità media di queste difficoltà? (selezionare)*

- ○ Difficoltà leggere
- ○ Difficoltà moderate
- ○ Difficoltà forti

Frequenza (fase acuta): *Con quale frequenza si presentano queste difficoltà? (selezionare)*
Frequenza (fase sub-acuta/cronica): *Con quale frequenza si sono presentate queste difficoltà negli ultimi 7 giorni? (selezionare)*

- ○ Raramente: meno di una volta al giorno
- ○ Spesso : da una a più volte al giorno
- ○ Continuamente : tutto il tempo

Se Raramente o Spesso:
Queste difficoltà sono più marcate in un momento specifico della giornata (per esempio la mattina al risveglio, a fine mattinata, a fine giornata o durante la notte)?

Commenti dello sperimentatore:
Es. Annotare se le risposte del paziente sembrano inventate e se si indicare per quale/i domanda/e

Altre sensazioni riportate spontaneamente dal paziente
Specificare per ogni sensazione/impressione se si riferisce all'arto ipsilesionale o controlesionale.

ARTO PIÙ COLPITO	3<br>ANOSODIAFORIA DELL'EMIPLEGIA

VAS CONTRA.3 da eseguire esclusivamente se la risposta a CONTRA 2 è minore di 14 (diversa dal minimo).

Alto (0)	*Queste difficoltà hanno un impatto emotivo su di me*<br>*Ripetere che si fa riferimento alle difficoltà motorie indagate precedentemente*
Basso (14)	*Queste difficoltà non hanno un impatto emotivo su di me*<br>*Ripetere che si fa riferimento alle difficoltà motorie indagate precedentemente*

Se è presente un impatto emotivo (punteggio < 14)

Intensità (fase acuta): *Con quale intensità, in media, è emotivamente colpita/o da queste difficoltà? (selezionare)*
Intensità (fase sub-acuta): *Negli ultimi 7 giorni, con quale intensità, in media, è stato emotivamente colpita/o da queste difficoltà? (selezionare)*

- ○ Colpita/o leggermente
- ○ Colpita/o moderatamente
- ○ Colpita/o severamente

Frequenza (fase acuta): *Con quale frequenza si sente emotivamente colpita/o da queste difficoltà? (selezionare)*
Frequenza (fase sub-acuta/cronica): *Con quale frequenza si è sentito emotivamente colpita/o da queste difficoltà negli ultimi 7 giorni? (selezionare)*

- ○ Raramente: meno di una volta al giorno
- ○ Spesso : da una a più volte al giorno
- ○ Continuamente : tutto il tempo

Se Raramente o Spesso:
Questa sensazione emotiva è più pronunciata in un momento particolare della giornata (per esempio la mattina al risveglio, a fine mattinata, a fine giornata o durante la notte)?

Commenti dello sperimentatore:
Es. Annotare se le risposte del paziente sembrano inventate e se si indicare per quale/i domanda/e

Altre sensazioni riportate spontaneamente dal paziente
Specificare per ogni sensazione/impressione se si riferisce all'arto ipsilesionale o controlesionale.

ARTO PIÙ COLPITO	4<br>EMI-ASOMATOAGNOSIA

Alto (14)	Ho l'impressione che questo braccio/ questa mano non faccia parte del mio corpo, che non mi appartenga
Basso (0)	Ho l'impressione che questo braccio/ questa mano faccia parte del mio corpo, che mi appartenga

Se il punteggio è > 0

Intensità (fase acuta): *Quale è l'intensità media di questa impressione? (selezionare)*
Intensità (fase sub-acuta): *Negli ultimi 7 giorni, quale è stata l'intensità media di questa impressione? (selezionare)*

- O Impressione lieve
- O Impressione moderata
- O Impressione forte

Frequenza (fase acuta): *Con quale frequenza si presenta questa impressione? (selezionare)*
Frequenza (fase sub-acuta/cronica): *Con quale frequenza si è presentata questa impressione negli ultimi 7 giorni? (selezionare)*

- O Raramente: meno di una volta al giorno
- O Spesso : da una a più volte al giorno
- O Continuamente : tutto il tempo

Se Raramente o Spesso:
Questa impressione è più marcata in un momento specifico della giornata (per esempio la mattina al risveglio, a fine mattinata, a fine giornata o durante la notte)?

Commenti dello sperimentatore:
Es. Annotare se le risposte del paziente sembrano inventate e se si indicare per quale/i domanda/e

Altre sensazioni riportate spontaneamente dal paziente
Specificare per ogni sensazione/impressione se si riferisce all'arto ipsilesionale o controlesionale.

ARTO PIÙ COLPITO	5.a. SOMATOPARAFRENIA (1/2)

Alto (14)	Ho l'impressione che questo braccio/questa mano appartenga a qualcun altro
Basso (0)	Non ho l'impressione che questo braccio/questa mano appartenga a qualcun altro

Se il punteggio è > 0

Se ha l'impressione che il braccio/la mano appartenga a qualcun altro, precisare: "a chi appartiene questo braccio/questa mano?"

Intensità (fase acuta): *Quale è l'intensità media di questa impressione? (selezionare)*
Intensità (fase sub-acuta): *Negli ultimi 7 giorni, quale è stata l'intensità media di questa impressione? (selezionare)*

- ○ Impressione lieve
- ○ Impressione moderata
- ○ Impressione forte

Frequenza (fase acuta): *Con quale frequenza si presenta questa impressione? (selezionare)*
Frequenza (fase sub-acuta/cronica): *Con quale frequenza si è presentata questa impressione negli ultimi 7 giorni? (selezionare)*

- ○ Raramente: meno di una volta al giorno
- ○ Spesso : da una a più volte al giorno
- ○ Continuamente : tutto il tempo

Se Raramente o Spesso:
Questa impressione è più marcata in un momento specifico della giornata (per esempio la mattina al risveglio, a fine mattinata, a fine giornata o durante la notte)?

Commenti dello sperimentatore:
Es. Annotare se le risposte del paziente sembrano inventate e se si indicare per quale/i domanda/e

Altre sensazioni riportate spontaneamente dal paziente
Specificare per ogni sensazione/impressione se si riferisce all'arto ipsilesionale o controlesionale.

ARTO PIÙ COLPITO	5.b. SOMATOPARAFRENIA (2/2)

Alto (14)	Ho l'impressione che questo braccio/questa mano corrisponda a qualcosa di non umano<br>*Per esempio, un oggetto.*
Basso (0)	Non ho l'impressione che questo braccio/questa mano corrisponda a qualcosa di non umano

Se il punteggio è > 0

Se ha l'impressione che il braccio/la mano corrisponda a qualcosa di non umano, precisare: "a che cosa corrisponde questo braccio/questa mano?"

Intensità (fase acuta): *Quale è l'intensità media di questa impressione? (selezionare)*
Intensità (fase sub-acuta): *Negli ultimi 7 giorni, quale è stata l'intensità media di questa impressione? (selezionare)*

- O Impressione lieve
- O Impressione moderata
- O Impressione forte

Frequenza (fase acuta): *Con quale frequenza si presenta questa impressione? (selezionare)*
Frequenza (fase sub-acuta/cronica): *Con quale frequenza si è presentata questa impressione negli ultimi 7 giorni? (selezionare)*

- O Raramente: meno di una volta al giorno
- O Spesso : da una a più volte al giorno
- O Continuamente : tutto il tempo

Se Raramente o Spesso:
Questa impressione è più marcata in un momento specifico della giornata (per esempio la mattina al risveglio, a fine mattinata, a fine giornata o durante la notte)?

Commenti dello sperimentatore:
Es. Annotare se le risposte del paziente sembrano inventate e se si indicare per quale/i domanda/e

Altre sensazioni riportate spontaneamente dal paziente
Specificare per ogni sensazione/impressione se si riferisce all'arto ipsilesionale o controlesionale.

ARTO PIÙ COLPITO	6<br>PERSONIFICAZIONE DELL'ARTO AFFETTO

Alto (14)	Ho l'impressione che questo braccio/questa mano abbia una propria personalità o identità<br>Per esempio: mi capita di dargli un nome, un soprannome o un diminutivo.
Basso (0)	Non ho l'impressione che questo braccio/questa mano abbia una propria personalità o identità

Se il punteggio è > 0

Se ha l'impressione che il braccio/la mano abbia una personalità o identità, precisare quale personalità/identità:

Intensità (fase acuta): *Quale è l'intensità media di questa impressione? (selezionare)*
Intensità (fase sub-acuta): *Negli ultimi 7 giorni, quale è stata l'intensità media di questa impressione? (selezionare)*

- O Impressione lieve
- O Impressione moderata
- O Impressione forte

Frequenza (fase acuta): *Con quale frequenza si presenta questa impressione? (selezionare)*
Frequenza (fase sub-acuta/cronica): *Con quale frequenza si è presentata questa impressione negli ultimi 7 giorni? (selezionare)*

- O Raramente: meno di una volta al giorno
- O Spesso : da una a più volte al giorno
- O Continuamente : tutto il tempo

Se Raramente o Spesso:
Questa impressione è più marcata in un momento specifico della giornata (per esempio la mattina al risveglio, a fine mattinata, a fine giornata o durante la notte)?

Commenti dello sperimentatore:
Es. Annotare se le risposte del paziente sembrano inventate e se si indicare per quale/i domanda/e

Altre sensazioni riportate spontaneamente dal paziente
Specificare per ogni sensazione/impressione se si riferisce all'arto ipsilesionale o controlesionale.

ARTO PIÙ COLPITO	7.a. ILLUSIONE DI CAMBIAMENTO DELLE CARATTERISTICHE FISICHE (TEMPERATURA)

Alto (14)	Ho l'impressione che questo braccio/questa mano abbia cambiato temperatura<br>*Ovvero lo percepisce più caldo/a o più freddo/a*<br>*Nota (fase acuta) : si fa riferimento a un cambiamento dopo l'ictus/a partire da questo ricovero.*<br>*Nota (fase sub-acuta) : si fa riferimento a un cambiamento dopo l'ictus/a partire da questo ricovero, e percepito negli ultimi 7 giorni.*
Basso (0)	Ho l'impressione che questo braccio/questa mano non abbia cambiato temperatura

Se il punteggio è > 0

Se ha l'impressione che questo braccio/questa mano abbia cambiato temperatura, precisare se è:

○ Più caldo/a
○ Più freddo/a

A quale parte del corpo ci si riferisce? (indicare la/le risposta/e corrispondente/i) :

☐ Braccio ☐ Avambaccio ☐ Mano ☐ Dita

Precisare, se necessario:

Intensità (fase acuta): *Quale è l'intensità media di questa impressione? (selezionare)*
Intensità (fase sub-acuta): *Negli ultimi 7 giorni, quale è stata l'intensità media di questa impressione? (selezionare)*

○ Impressione lieve
○ Impressione moderata
○ Impressione forte

Frequenza (fase acuta): *Con quale frequenza si presenta questa impressione? (selezionare)*
Frequenza (fase sub-acuta/cronica): *Con quale frequenza si è presentata questa impressione negli ultimi 7 giorni? (selezionare)*

○ Raramente: meno di una volta al giorno
○ Spesso : da una a più volte al giorno
○ Continuamente : tutto il tempo

Se Raramente o Spesso:
Questa impressione è più marcata in un momento specifico della giornata (per esempio la mattina al risveglio, a fine mattinata, a fine giornata o durante la notte)?

Commenti dello sperimentatore:
Es. Annotare se le risposte del paziente sembrano inventate e se si indicare per quale/i domanda/e

Altre sensazioni riportate spontaneamente dal paziente

Specificare per ogni sensazione/impressione se si riferisce all'arto ipsilesionale o controlesionale.

ARTO PIÙ COLPITO	**7.b.** **ILLUSIONE DI CAMBIAMENTO DELLE CARATTERISTICHE FISICHE (PESO)**

Alto (14)	Ho l'impressione che questo braccio/questa mano abbia cambiato peso<br>*Ovvero lo percepisce più leggero/a o più pesante*<br>*Nota (fase acuta) : si fa riferimento a un cambiamento dopo l'ictus/a partire da questo ricovero.*<br>*Nota (fase sub-acuta) : si fa riferimento a un cambiamento dopo l'ictus/a partire da questo ricovero, e percepito negli ultimi 7 giorni.*
Basso (0)	Ho l'impressione che questo braccio/questa mano non abbia cambiato peso

Se il punteggio è > 0

Se ha l'impressione che questo braccio/questa mano abbia cambiato peso, precisare se è:

○ Più pesante
○ Più leggero/a

A quale parte del corpo ci si riferisce? (indicare la/le risposta/e corrispondente/i) :

☐ Braccio ☐ Avambaccio ☐ Mano ☐ Dita

Precisare, se necessario:

Intensità (fase acuta): *Quale è l'intensità media di questa impressione? (selezionare)*
Intensità (fase sub-acuta): *Negli ultimi 7 giorni, quale è stata l'intensità media di questa impressione? (selezionare)*

○ Impressione lieve
○ Impressione moderata
○ Impressione forte

Frequenza (fase acuta): *Con quale frequenza si presenta questa impressione? (selezionare)*
Frequenza (fase sub-acuta/cronica): *Con quale frequenza si è presentata questa impressione negli ultimi 7 giorni? (selezionare)*

○ Raramente: meno di una volta al giorno
○ Spesso : da una a più volte al giorno
○ Continuamente : tutto il tempo

Se Raramente o Spesso:
Questa impressione è più marcata in un momento specifico della giornata (per esempio la mattina al risveglio, a fine mattinata, a fine giornata o durante la notte)?

Commenti dello sperimentatore:
Es. Annotare se le risposte del paziente sembrano inventate e se si indicare per quale/i domanda/e

Altre sensazioni riportate spontaneamente dal paziente

Specificare per ogni sensazione/impressione se si riferisce all'arto ipsilesionale o controlesionale.

ARTO PIÙ COLPITO	7.c. ILLUSIONE DI CAMBIAMENTO DELLE CARATTERISTICHE FISICHE (LUNGHEZZA)

Alto (14)	Ho l'impressione che questo braccio/questa mano abbia cambiato lunghezza<br>*Ovvero lo percepisce più lungo/a o più corto/a*<br>*Nota (fase acuta) : si fa riferimento a un cambiamento dopo l'ictus/a partire da questo ricovero.*<br>*Nota (fase sub-acuta) : si fa riferimento a un cambiamento dopo l'ictus/a partire da questo ricovero, e percepito negli ultimi 7 giorni.*
Basso (0)	Ho l'impressione che questo braccio/questa mano non abbia cambiato lunghezza

Se il punteggio è > 0

Se ha l'impressione che questo braccio/questa mano abbia cambiato lunghezza, precisare se è:

○ Più lungo/a
○ Più corto/a

A quale parte del corpo ci si riferisce? (indicare la/le risposta/e corrispondente/i) :

☐ Braccio ☐ Avambaccio ☐ Mano ☐ Dita

Precisare, se necessario:

Intensità (fase acuta): *Quale è l'intensità media di questa impressione? (selezionare)*
Intensità (fase sub-acuta): *Negli ultimi 7 giorni, quale è stata l'intensità media di questa impressione? (selezionare)*

○ Impressione lieve
○ Impressione moderata
○ Impressione forte

Frequenza (fase acuta): *Con quale frequenza si presenta questa impressione? (selezionare)*
Frequenza (fase sub-acuta/cronica): *Con quale frequenza si è presentata questa impressione negli ultimi 7 giorni? (selezionare)*

○ Raramente: meno di una volta al giorno
○ Spesso : da una a più volte al giorno
○ Continuamente : tutto il tempo

Se Raramente o Spesso:
Questa impressione è più marcata in un momento specifico della giornata (per esempio la mattina al risveglio, a fine mattinata, a fine giornata o durante la notte)?

Commenti dello sperimentatore:
Es. Annotare se le risposte del paziente sembrano inventate e se si indicare per quale/i domanda/e

Altre sensazioni riportate spontaneamente dal paziente

Specificare per ogni sensazione/impressione se si riferisce all'arto ipsilesionale o controlesionale.

ARTO PIÙ COLPITO	8<br>MOVIMENTI ILLUSORI

Alto (14)	*Ho l'impressione che questo braccio/questa mano si muova anche se in realtà non si muove*<br>*Per esempio: ho l'impressione che questo braccio/questa mano si muova, ma quando lo/la guardo per verificare, in realtá non si sta muovendo.*
Basso (0)	*Ho l'impressione che questo braccio/questa mano si muova unicamente quando effettivamente si muove nella realtá*

Se il punteggio è > 0

Se ha l'impressione di movimenti illusori, specificare (selezionare la/le risposte corrispondente/i):

☐ Questa impressione è una sensazione sensoriale (lei sperimenta una sensazione di movimento)

☐ Questa sensazione è una rappresentazione mentale (lei visualizza il movimento nella sua mente)

☐ Questa impressione avviene quando lei ha intenzione di mettere il braccio/la mano in movimento

☐ Questa impressione di movimento avviene quando lei non ha intenzione di mettere in movimento il braccio/la mano

Precisazioni e/o commenti:

Intensità (fase acuta): *Quale è l'intensità media di questa impressione? (selezionare)*
Intensità (fase sub-acuta): *Negli ultimi 7 giorni, quale è stata l'intensità media di questa impressione? (selezionare)*

○ Impressione lieve
○ Impressione moderata
○ Impressione forte

Frequenza (fase acuta): *Con quale frequenza si presenta questa impressione? (selezionare)*
Frequenza (fase sub-acuta/cronica): *Con quale frequenza si è presentata questa impressione negli ultimi 7 giorni? (selezionare)*

○ Raramente: meno di una volta al giorno
○ Spesso : da una a più volte al giorno
○ Continuamente : tutto il tempo

Se Raramente o Spesso:
Questa impressione è più marcata in un momento specifico della giornata (per esempio la mattina al risveglio, a fine mattinata, a fine giornata o durante la notte)?

Commenti dello sperimentatore:
Es. Annotare se le risposte del paziente sembrano inventate e se si indicare per quale/i domanda/e

Altre sensazioni riportate spontaneamente dal paziente

Specificare per ogni sensazione/impressione se si riferisce all'arto ipsilesionale o controlesionale.

ARTO PIÙ COLPITO	9<br>ARTO IN SOVRANNUMERO/SOTTONUMERO

Alto (14)	Ho l'impressione di avere più di due braccia e/o di due mani o ho l'impressione di avere meno di due braccia e/o due mani
Basso (0)	Ho l'impressione che il mio corpo sia costitutito da due braccia e due mani

Se il punteggio è > 0

Se ha l'impressione di avere più o meno di due braccia e/o due mani, specificare quante braccia/mani, e dove queste sono situate (zona del corpo e dimensione)?

Intensità (fase acuta): *Quale è l'intensità media di questa impressione? (selezionare)*
Intensità (fase sub-acuta): *Negli ultimi 7 giorni, quale è stata l'intensità media di questa impressione? (selezionare)*

○ Impressione lieve
○ Impressione moderata
○ Impressione forte

Frequenza (fase acuta): *Con quale frequenza si presenta questa impressione?   (selezionare)*
Frequenza (fase sub-acuta/cronica): *Con quale frequenza si è presentata questa impressione negli ultimi 7 giorni? (selezionare)*

○ Raramente: meno di una volta al giorno
○ Spesso : da una a più volte al giorno
○ Continuamente : tutto il tempo

Se Raramente o Spesso:
Questa impressione è più marcata in un momento specifico della giornata (per esempio la mattina al risveglio, a fine mattinata, a fine giornata o durante la notte)?

Commenti dello sperimentatore:
Es. Annotare se le risposte del paziente sembrano inventate e se si indicare per quale/i domanda/e

Altre sensazioni riportate spontaneamente dal paziente
Specificare per ogni sensazione/impressione se si riferisce all'arto ipsilesionale o controlesionale.

ARTO PIÙ COLPITO	**10** **ARTO SUPERIORE DISTACCATO DAL RESTO DEL CORPO**

Alto (14)	Ho l'impressione che questo braccio/questa mano sia distaccato/a dal resto del mio corpo *(il mio corpo mi sembra diviso)*
Basso (0)	Non ho l'impressione che questo braccio/questa mano sia distaccato/a dal resto del mio corpo

Se il punteggio è > 0

Se ha l'impressione che il corpo sia diviso, descriva questa impressione:

Intensità (fase acuta): *Quale è l'intensità media di questa impressione? (selezionare)*
Intensità (fase sub-acuta): *Negli ultimi 7 giorni, quale è stata l'intensità media di questa impressione? (selezionare)*

- O Impressione lieve
- O Impressione moderata
- O Impressione forte

Frequenza (fase acuta): *Con quale frequenza si presenta questa impressione? (selezionare)*
Frequenza (fase sub-acuta/cronica): *Con quale frequenza si è presentata questa impressione negli ultimi 7 giorni? (selezionare)*

- O Raramente: meno di una volta al giorno
- O Spesso : da una a più volte al giorno
- O Continuamente : tutto il tempo

Se Raramente o Spesso:
Questa impressione è più marcata in un momento specifico della giornata (per esempio la mattina al risveglio, a fine mattinata, a fine giornata o durante la notte)?

Commenti dello sperimentatore:
Es. Annotare se le risposte del paziente sembrano inventate e se si indicare per quale/i domanda/e

Altre sensazioni riportate spontaneamente dal paziente
Specificare per ogni sensazione/impressione se si riferisce all'arto ipsilesionale o controlesionale.

ARTO PIÙ COLPITO	**11** **MISOPLEGIA**

Alto (14)	Ho un atteggiamento avverso nei confronti di questo braccio/questa mano<br>*Per esempio: provo rabbia, disprezzo, aggressivitá (verbale e/o fisica) o odio per questo braccio/questa mano.*
Basso (0)	Non ho un atteggiamento avverso nei confronti di questo braccio/questa mano

Se il punteggio è > 0

Se ha un atteggiamento avverso, come si esprime?

a) Attraverso un sentimento

Per esempio: provo rabbia, disprezzo o odio verso questo braccio o questa mano.

b) Attraverso un comportamento

Per esempio: ho la tendenza a criticare negativamente o ad insultare questo braccio o questa mano, a spingerlo, colpirlo, graffiarlo o morderlo.

Intensità (fase acuta): *Con quale intensità, in media, si manifesta questo atteggiamento avverso? (selezionare)*
Intensità (fase sub-acuta): *Negli ultimi 7 giorni, con quale intensità, in media, questo atteggiamento avverso si è manifestato? (selezionare)*

○ Leggermente
○ Moderatamente
○ Fortemente / Severamente

Frequenza (fase acuta): *Con quale frequenza si manifesta questo atteggiamento avverso? (selezionare)*
Frequenza (fase sub-acuta/cronica): *Con quale frequenza questo atteggiamento avverso si è manifestato negli ultimi 7 giorni? (selezionare)*

○ Raramente: meno di una volta al giorno
○ Spesso : da una a più volte al giorno
○ Continuamente : tutto il tempo

Se Raramente o Spesso:
Questo attegiamento è più marcato in un momento specifico della giornata (per esempio la mattina al risveglio, a fine mattinata, a fine giornata o durante la notte)?

Commenti dello sperimentatore:
Es. Annotare se le risposte del paziente sembrano inventate e se si indicare per quale/i domanda/e

Altre sensazioni riportate spontaneamente dal paziente

Specificare per ogni sensazione/impressione se si riferisce all'arto ipsilesionale o controlesionale.

ARTO PIÙ COLPITO	12 MOVIMENTI INVOLONTARI

Alto (14)	*Questo braccio/questa mano fa dei movimenti involontari, non desiderati*<br>*Per esempio: questa mano si muove quando io non ho deciso di metterla in movimento.*
Basso (0)	*I movimenti di questo braccio/ questa mano sono volontari*<br>*Per esempio: questo mano si muove quando decido di metterla in movimento.*

Se il punteggio è > 0

Se questo braccio/questa mano ha un comportamento motorio involontario, specificare se presenta (segnare la /le risposte corrispondenti):

☐ Movimenti di sollevamento (il braccio /la mano ha la tendenza a sollevarsi verso l'alto).
☐ Movimenti di prensione, manipolazione o afferamento di oggetti (la mano ha la tendenza a prendere, manipolare o afferrare gli oggetti).
☐ Movimenti di conflitto tra le due mani: una mano esegue un'attività e l'altra mano disturba l'attività in corso eseguendo per esempio un'attività opposta (per esempio, una mano abbottona una camicia e l'altra mano sbottona la stessa camicia il momento successivo)
☐ * Movimenti (atassici) che mancano di precisione (nel momento di prendere un oggetto, la mano si avvicina all'obiettivo richiedendo di aggiustare il movimento di presa rispetto all'oggetto).
☐ * Movimenti maldestri o interrotti a causa della debolezza muscolare (ad esempio l'oggetto cade per via della mancanza di forza).
☐ Altro (se altro, specificare sotto).

** questi suggerimenti non sono considerati come movimenti involontari ma dovrebbero comunque essere segnati se presenti.*

Precisazioni e/o commenti:

Intensità (fase acuta): *Quale è l'intensità media di questi movimenti involontari? (selezionare)*
Intensità (fase sub-acuta): *Negli ultimi 7 giorni, quale è stata l'intensità media di questi movimenti involontari? (selezionare)*

○ Movimenti involontari lievi
○ Movimenti involontari moderati
○ Movimenti involontari forti

Frequenza (fase acuta): *Con quale frequenza si presentano questi movimenti involontari? (selezionare)*
Frequenza (fase sub-acuta/cronica): *Con quale frequenza si sono presentati questi movimenti involontari negli ultimi 7 giorni? (selezionare)*

○ Raramente: meno di una volta al giorno
○ Spesso : da una a più volte al giorno
○ Continuamente : tutto il tempo

Se Raramente o Spesso:
Questi movimenti involontari sono più marcati in un momento specifico della giornata (per esempio la mattina al risveglio, a fine mattinata, a fine giornata o durante la notte)?

Commenti dello sperimentatore:

Es. Annotare se le risposte del paziente sembrano inventate e se si indicare per quale/i domanda/e

Altre sensazioni riportate spontaneamente dal paziente

Specificare per ogni sensazione/impressione se si riferisce all'arto ipsilesionale o controlesionale.

ARTO MENO COLPITO	1<br>ANOSOGNOSIA DELL'EMIPLEGIA

Alto (0)	*Ho difficoltà a muovere questo braccio/questa mano*
Basso (14)	*Non ho difficoltà a muovere questo braccio/questa mano*

Se è presente una difficoltà (punteggio < 14)

Intensità (fase acuta): *Quale è l'intensità media di queste difficoltà? (selezionare)*
Intensità (fase sub-acuta): *Negli ultimi 7 giorni, quale è stata l'intensità media di queste difficoltà? (selezionare)*

- ○ Difficoltà leggere
- ○ Difficoltà moderate
- ○ Difficoltà forti

Frequenza (fase acuta): *Con quale frequenza si presentano queste difficoltà? (selezionare)*
Frequenza (fase sub-acuta/cronica): *Con quale frequenza si sono presentate queste difficoltà negli ultimi 7 giorni? (selezionare)*

- ○ Raramente: meno di una volta al giorno
- ○ Spesso : da una a più volte al giorno
- ○ Continuamente : tutto il tempo

Se Raramente o Spesso:
Queste difficoltá sono più marcate in un momento specifico della giornata (per esempio la mattina al risveglio, a fine mattinata, a fine giornata o durante la notte)?

Commenti dello sperimentatore:

Es. Annotare se le risposte del paziente sembrano inventate e se si indicare per quale/i domanda/e

Altre sensazioni riportate spontaneamente dal paziente

Specificare per ogni sensazione/impressione se si riferisce all'arto ipsilesionale o controlesionale.

ARTO MENO COLPITO	2<br>EMIASOMATOGNOSIA

Alto (14)	Ho l'impressione che questo braccio/ questa mano non faccia parte del mio corpo, che non mi appartenga
Basso (0)	Ho l'impressione che questo braccio/ questa mano faccia parte del mio corpo, che mi appartenga

Se il punteggio è > 0

Intensità (fase acuta): *Quale è l'intensità media di questa impressione? (selezionare)*
Intensità (fase sub-acuta): *Negli ultimi 7 giorni, quale è stata l'intensità media di questa impressione? (selezionare)*

- O Impressione lieve
- O Impressione moderata
- O Impressione forte

Frequenza (fase acuta): *Con quale frequenza si presenta questa impressione? (selezionare)*
Frequenza (fase sub-acuta/cronica): *Con quale frequenza si è presentata questa impressione negli ultimi 7 giorni? (selezionare)*

- O Raramente: meno di una volta al giorno
- O Spesso : da una a più volte al giorno
- O Continuamente : tutto il tempo

Se Raramente o Spesso:
Questa impressione è più marcata in un momento specifico della giornata (per esempio la mattina al risveglio, a fine mattinata, a fine giornata o durante la notte)?

Commenti dello sperimentatore:
Es. Annotare se le risposte del paziente sembrano inventate e se si indicare per quale/i domanda/e

Altre sensazioni riportate spontaneamente dal paziente
Specificare per ogni sensazione/impressione se si riferisce all'arto ipsilesionale o controlesionale.

ARTO MENO COLPITO	3 SOMATOPARAFRENIA

Alto (14)	Ho l'impressione che questo braccio/questa mano appartenga a qualcun'altro
Basso (0)	Non ho l'impressione che questo braccio/questa mano appartenga a qualcun'altro

Se il punteggio è > 0

Se ha l'impressione che il braccio/la mano appartenga a qualcun altro, precisare: "a chi appartiene questo braccio/questa mano?"

Intensità (fase acuta): *Quale è l'intensità media di questa impressione? (selezionare)*
Intensità (fase sub-acuta): *Negli ultimi 7 giorni, quale è stata l'intensità media di questa impressione? (selezionare)*

- ○ Impressione lieve
- ○ Impressione moderata
- ○ Impressione forte

Frequenza (fase acuta): *Con quale frequenza si presenta questa impressione? (selezionare)*
Frequenza (fase sub-acuta/cronica): *Con quale frequenza si è presentata questa impressione negli ultimi 7 giorni? (selezionare)*

- ○ Raramente: meno di una volta al giorno
- ○ Spesso : da una a più volte al giorno
- ○ Continuamente : tutto il tempo

Se Raramente o Spesso:
Questa impressione è più marcata in un momento specifico della giornata (per esempio la mattina al risveglio, a fine mattinata, a fine giornata o durante la notte)?

Commenti dello sperimentatore:
Es. Annotare se le risposte del paziente sembrano inventate e se si indicare per quale/i domanda/e

Altre sensazioni riportate spontaneamente dal paziente
Specificare per ogni sensazione/impressione se si riferisce all'arto ipsilesionale o controlesionale.

ALTRE SENSAZIONI

Ha altre sensazioni relative alle braccia/mani che non sono state menzionate?
Specificare per ogni sensazione/impressione se si riferisce all'arto ipsilesionale o controlesionale.

Commenti dello sperimentatore:
Es. Annotare se le risposte del paziente sembrano inventate e se si, indicare quale/i sensazione/i
